# The Infectivity and Pathogenicity Characteristics of a Recombinant Porcine Epidemic Diarrhea Virus, CHFJFQ

**DOI:** 10.3390/v17030401

**Published:** 2025-03-12

**Authors:** Zhihua Feng, Heng Zhao, Zhaolong Li, Minhua Lin, Weili Huang, Chuancheng Liu, Yangkun Shen, Qi Chen

**Affiliations:** 1 Key Laboratory of OptoElectronic Science and Technology for Medicine of Ministry of Education, Fujian Normal University, Fuzhou 350117, China; fengzhihua@fjnu.edu.cn; 2 Fujian Key Laboratory of Innate Immune Biology, Biomedical Research Center of South China, Fujian Normal University Qishan Campus, Fuzhou 350117, China; qbx20200151@yjs.fjnu.edu.cn (H.Z.); 18150193086@163.com (M.L.); qsx20211392@student.fjnu.edu.cn (W.H.); qsx20211374@student.fjnu.edu.cn (C.L.); 3 College of Life Science, Fujian Normal University Qishan Campus, Fuzhou 350117, China; 4 College of Photonic and Electronic Engineering, Fujian Normal University, Fuzhou 350117, China; 5 Institute of Animal Husbandry and Veterinary Medicine, Fujian Academy of Agricultural Sciences, Fuzhou 350013, China; lizhaolong@faas.cn

**Keywords:** PEDV, origin, infection, cross-species transmission

## Abstract

Porcine epidemic diarrhea virus (PEDV) presents a substantial challenge to the global swine industry. However, the origin, host range, and potential cross-species transmission of PEDV remain poorly understood. This study characterizes a novel PEDV strain, CHFJFQ, isolated from diarrheic piglets in Fuqing, Fujian, China. Through sequencing and phylogenetic analysis, we determined that CHFJFQ belongs to the GIIa subgroup and is a recombinant with CH/HNXX/2016 as the major parent and NW17 as the minor parent. Compared to CV777, CHFJFQ exhibits multiple base deletions and insertions across the 5′UTR, ORF1a/b, S, and ORF3 genes. Phylogenetic analysis indicates shared ancestry with bat coronaviruses, though a direct zoonotic origin remains uncertain. Interestingly, CHFJFQ demonstrated its ability to infect human and mouse cell lines in vitro and, more significantly, caused in vivo infection in both pigs and mice. The primary target organs were the intestines, lungs, and spleen, resulting in 100% mortality in suckling piglets. PEDV CHFJFQ was detected in mouse tissues, but no clinical signs were observed, indicating limited cross-species pathogenicity. Overall, these findings offer crucial insights into the epidemiology, genetics, infectivity, and pathogenicity of PEDV and provide valuable information for vaccine development.

## 1. Introduction

**Figure 1:**
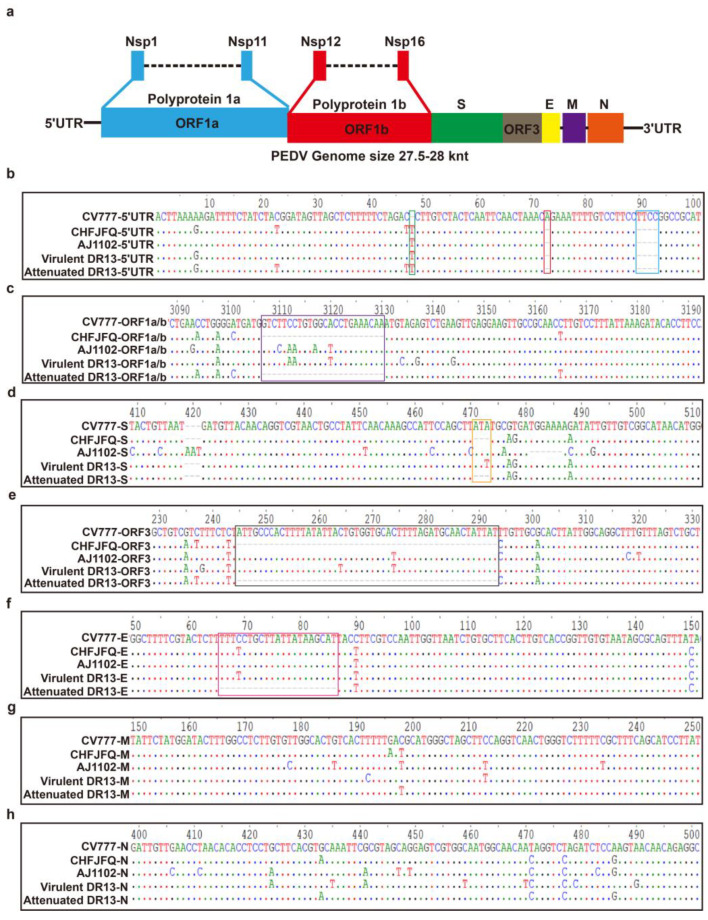
Nucleotide deletions and insertions in the genome of PEDV CHFJFQ compared to CV777, AJ1102, and DR13. (**a**) Blue strip: Polyprotein 1a and non-structural proteins 1–11; red strip: polyprotein 1b and non-structural proteins 12–16; green strip: spike protein (S); brown: ORF3; yellow strip: envelope protein; purple strip: membrane protein; orange strip: nucleocapsid protein. (**b**) Compared to the CV777 strain, 5 nucleotides were deleted, and 1 nucleotide was inserted in the 5′ UTR of CHFJFQ, AJ1102, virulent DR13, and attenuated DR13. (**c**) Compared to the CV777 strain, a 24-nucleotide deletion was observed in the ORF1a/b region of both the CHFJFQ and attenuated DR13 strains. (**d**) Compared to the CV777 strain, CHFJFQ and attenuated DR13 strains showed a 3-nucleotide deletion in the S gene, while AJ1102 exhibited a 6-nucleotide deletion and 3-nucleotide insertion in the S gene. (**e**) Based on the CV777 strain, a 49-nucleotide deletion was observed in the ORF3 region of both CHFJFQ and attenuated DR13. (**f**) Compared to CV777, CHFJFQ, AJ1102, and virulent DR13 strains, a 21-nucleotide deletion was observed in the E gene of the attenuated DR13 strain. (**g**,**h**) No nucleotide deletions or insertions were observed in the M or N gene of the CV777, CHFJFQ, AJ1102, virulent DR13, and attenuated DR13 strains. Sequence alignment was performed using BioEdit software (Version 7.0.9.0), with ClustalW multiple alignment selected (Full Multiple Alignment, Bootstrap NJ Tree, number of bootstraps set to 1000).

Porcine epidemic diarrhea (PED) poses a substantial threat to the swine industry, characterized as an acute and highly contagious enteric disease affecting pigs of all ages, caused by the porcine epidemic diarrhea virus (PEDV) [1]. PEDV is classified within the Alphacoronavirus genus as an enveloped, positive-sense, single-stranded RNA virus. The PEDV genome, typical of coronaviruses, is a linear, non-segmented, ~28 kbp RNA molecule with a 5′ cap and 3′ poly(A) tail, packaged within a helical nucleocapsid [2]. This genome encodes at least seven open reading frames (ORFs), encoding four structural proteins and 17 non-structural proteins [3,4]. From 5′ to 3′, the genome is organized into a 5′ untranslated region (UTR), open reading frame 1a/b (ORF1a/b), genes for the spike (S), envelope (E), membrane (M), and nucleocapsid (N) proteins, a hypothetical accessory protein ORF3, the 3′ UTR, and a poly(A) tail (Figure 1a) [5].

ORF1a/b encodes polyprotein 1a (PP1a) and polyprotein 1b (PP1b), respectively, which undergo proteolytic cleavage to produce 16 non-structural proteins (nsp1–16) [3]. These nsps are critical for viral genome replication and play a significant role in immune evasion, thereby contributing to PEDV pathogenesis [6,7]. ORF3, an accessory protein associated with PEDV virulence, exhibits ion channel activity and plays a crucial role in promoting vesicle formation, inhibiting cell apoptosis, and enhancing viral replication [8,9]. Notably, ORF3 is prone to deletion or mutation during the cellular adaptation of PEDV through multiple passages in vitro [10]. Attenuated PEDV strains, such as DR13, KPED-9, and P-5V, harbor deletions of 49 or 51 nucleotides in the ORF3 gene, which are believed to contribute to their reduced pathogenicity [11,12]. The spike protein (S) is a 150–220 kDa glycoprotein located on the surface of PEDV and mediates viral attachment and entry into host cells [13]. Following trypsin cleavage, the S protein is processed into S1 and S2 subunits. The S1 subunit binds to cellular receptors during PEDV infection, while the S2 subunit facilitates fusion of the viral and cellular membranes, facilitating entry [14]. In addition, the S protein is an important inducer of anti-PEDV neutralizing antibodies in vivo [14]. High rates of mutation in the S gene makes it a valuable marker for assessing the genetic diversity of PEDV [15]. The envelope (E) protein, a small (approximately 7 kDa) structural component of PEDV, participates in viral particle maturation and release and modulates virus–host interactions. These modulations frequently manifest as cell stress, activation of the unfolded protein response (UPR), induction of apoptosis, and inflammation [16]. A key structural component of the PEDV virion, the membrane (M) protein (27–32 kDa) is the most abundant protein in the viral envelope and is essential for assembly and budding. The M protein also antagonizes the host’s innate immune system by blocking type I and type III interferon pathways, and it elicits the production of protective antibodies in pigs [17,18]. The PEDV nucleocapsid (N) protein, a highly conserved phosphoprotein residing in the endoplasmic reticulum, performs multiple crucial functions during the viral life cycle. These include regulating viral RNA synthesis, encapsidating the viral genome within the helical nucleocapsid, and participating in viral particle assembly [19]. Additionally, the N protein promotes viral proliferation by inducing ER stress, regulating the NF-κB signaling pathway, and modulating autophagy [20].

Initially identified in the United Kingdom in the 1970s, porcine epidemic diarrhea (PED) has since spread to numerous countries throughout Europe and Asia, causing substantial setbacks in the development of the pork industry [21]. The recent resurgence of PED is largely driven by PEDV mutant strains, particularly in China, highlighting the virus’s rapid evolution. Phylogenetic analysis of the S gene commonly differentiates PEDV into two main genotypes, GI and GII [22]. GI is further divided into two (a and b) or three (a, b, and c) subtypes. Similarly, GII encompasses subtypes a and b, with subsequent diversification of GIIa and GIIb into multiple lineages [5,15]. Importantly, genotype GII encompasses the majority of strains currently circulating in China, the United States, and other Asian countries and responsible for PEDV outbreaks. In comparison to traditional genotype I (GI) PEDV strains, most contemporary genotype II (GII) strains display multiple deletions and insertions within the spike (S) gene, which may account for the limited effectiveness of existing vaccines [23,24]. PEDV is capable of infecting swine of all ages, with diarrhea, vomiting, dehydration, enteritis, and emaciation being the most commonly observed clinical manifestations. Particularly vulnerable are neonatal piglets (one week of age or less), in which PEDV infection often results in mortality rates approaching 100% [25]. Consequently, due to the insufficient protection offered by vaccines derived from traditional strains, the development of novel vaccines predicated on a more comprehensive understanding of PEDV evolution is critically important.

Cross-species transmission remains a central concern in coronavirus research, given the viruses’ demonstrated capacity for adaptation and emergence in novel hosts. The ability of SARS-CoV-2 to infect species such as felines and rodents underscores this threat [26]. Although natural infection of species other than swine has not been documented for PEDV to date, its in vitro infectivity across a range of cell lines, including human (HEK 293, Huh-7, and MRC-5), monkey (Vero-CCL-81), bat (Tb1-Lu), and pig (PK15), is noteworthy [27]. Phylogenetic evidence suggests a possible origin of PEDV in bat coronaviruses, consistent with its ability to infect bat cells [28], mirroring patterns observed in alpha- and betacoronaviruses. This in vitro tropism of PEDV for human cells, coupled with its demonstrated capacity to infect diverse species, underscores the need for vigilance regarding potential cross-species transmission [29].

Between 2014 and 2018, porcine diarrhea outbreaks affected pig farms in Fuqing, Fujian Province, China. We isolated a novel PEDV strain, CHFJFQ. The PEDV CHFJFQ strain emerged in pig farms using standardized PEDV vaccines, coinciding with a devastating outbreak characterized by near-total (100%) diarrhea and >90% mortality in piglets. This stark vaccine failure indicates a potential escape from existing immunity. Moreover, CHFJFQ infection resulted in atypical lesions affecting multiple organs, including lung and kidney hemorrhage, and intestinal fluid accumulation. Intriguingly, we detected PEDV RNA in the lungs, contradicting prior reports. Given these unique features, we deemed it critical to investigate the genomic and infection characteristics of CHFJFQ to understand its implications for PEDV evolution, prevalence, and pathogenesis in China.

## 2. Materials and Methods

### 2.1. Cells, Viruses, and Antibodies

Human embryonic kidney (HEK) 293A cells, African green monkey kidney fibroblast cells (Vero), intestinal porcine epithelial cell line J2 cells (IPEC-J2), NCTC clone fibrosarcoma cells (L929), swine testis cells (ST), and porcine kidney 15 (PK15) cells were obtained from the American Type Culture Collection (Manassas, VA, USA). The cells were cultured in Dulbecco’s Modified Eagle’s Medium (DMEM) (SH30022.01, Cytiva, Shanghai, China) supplemented with 10% (*v*/*v*) fetal bovine serum (FBS) (10099-141C, Gibco, Vacaville, CA, USA), 10 μg/mL streptomycin, and 100 U/mL penicillin (S110JV, BasalMedia, Shanghai, China). The cells were maintained at 37 °C in a 5% CO_2_ atmosphere. The PEDV CHFJFQ strain was isolated in our laboratory in 2018 from intestinal tract samples of pigs with diarrhea in Fuqing, Fujian, China. Anti-PEDV N protein monoclonal antibody (SD-2-1) was purchased from Medgene Labs (Brookings, SD, USA). GAPDH (60004-1-Ig) antibody and Beta Actin recombinant antibody (81115-1-RR) were obtained from Proteintech (Wuhan, China). Additionally, IRDye^®^ 800CW donkey anti-mouse IgG secondary antibody (926-32212, LI-COR, Lincoln, NE, USA), Alexa Fluor™ 546 donkey anti-mouse IgG (H + L) (A10036, Invitrogen, Carlsbad, CA, USA), and highly cross-adsorbed secondary antibody (1832039, Invitrogen) were used.

### 2.2. Isolation and Genome Sequencing of PEDV CHFJFQ

In the winter of 2018, outbreaks of diarrhea occurred in several pig farms in Fuqing City, Fujian Province, China, despite routine PEDV vaccination. Diarrhea affected pigs of all ages. Approximately 5000 pigs were involved, with a near 100% morbidity rate and over 90% mortality rate in piglets. Intestinal tissue samples were collected from diarrheal piglets. Using RT-PCR, the PEDV genome was detected in these tissues; however, TGEV genome was not detected (Supplementary Figure S1). The positive PCR controls consisted of the plasmids pcDNA3.1-PEDV-S and pcDNA3.1-TGEV-S (containing the respective PEDV and TGEV S genes), while ddH_2_O served as the negative control. Given the high infection and mortality rates, we attempted viral isolation.

The intestinal samples were minced and diluted to 1 mL using PBS, followed by centrifugation at 12,000 rpm for 10 min. The supernatant was filtered through a 0.45 μm filter, and 1 mL of the filtrate was co-cultured with 5 × 10^6^ Vero cells. Trypsin was added to a final concentration of 10 μg/mL. Cell density and cytopathic effects were observed every 12 h. Cells were harvested once more than 80% cytopathic effects were observed and stored at −80 °C. PEDV CHFJFQ was used to infect Vero cells for 36 h (eight passages, MOI: 0.2). Total RNA was extracted using TRIzol™ (15596018, Thermo Fisher, Waltham, MA, USA) and reverse-transcribed into cDNA using the HiScript III RT SuperMix for quantitative real-time polymerase chain reaction (qPCR) (+gDNA wiper) (R323-01; Vazyme, Nanjing, China). The PEDV CHFJFQ genome was amplified using polymerase chain reaction (PCR) with DNA polymerase (2 × Phanta Max Master Mix (Dye Plus), P525-01, Vazyme). The PCR conditions were as follows: 95 °C for 5 min, followed by 35 cycles of 95 °C for 10 s, 58 °C for 30 s, and 72 °C for 1 min. Primers (Supplementary Table S1) were designed based on the PEDV-SX genome (GenBank accession KY420075.1). The PCR products were purified using the FastPure Gel DNA Extraction Mini Kit (DC301-01, Vazyme) and subsequently sequenced by Fuzhou Shangya Biotechnology Co., Ltd. (Fuzhou, China).

### 2.3. Reverse Transcription–Polymerase Chain Reaction (RT-PCR) and Agarose Gel Electrophoresis

Approximately 100 mg of intestinal tissue was collected, and total RNA was extracted using TRIzol™ reagent according to the manufacturer’s instructions. cDNA was synthesized from 1 μg of RNA using a HiScript III RT SuperMix for quantitative real-time polymerase chain reaction (qPCR) (+gDNA wiper). PCR amplification was performed using 200 ng of cDNA as template and a 2 × Phanta Max Master Mix (Dye Plus). The PCR cycling conditions were as follows: initial denaturation at 95 °C for 3 min; 35 cycles of denaturation at 95 °C for 30 s, annealing at 58 °C for 30 s, and extension at 72 °C for 30 s; and a final extension at 72 °C for 7 min. The following primer pairs were used for PCR amplification of the PEDV and TGEV S genes: PEDV-S-PCR-F: TGCCAATGTATTTGCCACT, PEDV-S-PCR-R: TGACAGTAGGAGGTAAAACAGCC, TGEV-S-PCR-F: AATGCCCTTAAATTGGCTTCT, TGEV-S-PCR-R: CCCTAACCTCGGCTTGTCTG. Following PCR, 10 μL of the PCR product was analyzed by agarose gel electrophoresis. Gels were prepared using a 1.0–1.5% (*w*/*v*) agarose concentration, and electrophoresis was conducted at 180 V for 12 min.

### 2.4. Transmission Electron Microscope Assay

Vero cells (5 × 10^5^ per well) were infected with PEDV (MOI = 0.01) and cultured in 6-well plates. At 48 h post-infection (hpi), the culture medium was discarded, and the cells were washed twice with PBS. The cells were then collected and fixed in 2.5% glutaraldehyde (G1102, Servicebio, Wuhan, China) at 4 °C for 2 h. After fixation, the cells were transferred to a 1.5 mL centrifuge tube. Transmission electron microscopy (TEM) sample preparation was carried out by Servicebio (Wuhan, China), and images were captured using a TEM platform (HITACHI, model H-7650).

### 2.5. PEDV Amplification and Titer Assays

The seventh-passage viruses were co-cultured with 5 × 10^6^ Vero cells at 37 °C, with trypsin added to the culture medium at a final concentration of 10 μg/mL. The culture supernatant and cells were harvested when the cytopathic effects exceeded 80% and stored at −80 °C. The cultures were then centrifuged at 3000 rpm for 5 min, and the supernatant was collected for titer determination. The titer was determined using the Spearman–Kärber method [30,31]. The Spearman–Kärber method is a widely recognized and validated technique for viral titer determination, particularly when dealing with cytopathic viruses like PEDV. Its reliability has been demonstrated in numerous virological studies, including those focused on coronaviruses. Briefly, serial ten-fold dilutions of the virus were co-cultured with Vero cells in 96-well plates. Specifically, 5000 Vero cells were seeded into each well of a 96-well plate containing 90 μL of DMEM. The first column of wells served as a virus-free control. Starting with the second column, 10 μL of the original viral stock was added to each well, resulting in a 10-fold dilution (10^−1^). The mixture was thoroughly mixed by pipetting, and then 10 μL was transferred to the next column of cells, creating a 100-fold dilution (10^−2^). This serial ten-fold dilution was repeated across the plate, reaching a final dilution of 10^−11^. The cytopathic effect (CPE) was monitored every 24 h until a clear endpoint was reached, indicated by a sufficient number of wells exhibiting no CPE. At this point, the experiment was terminated. The virus titer was calculated using the formula lgTCID50 = L − d (S − 0.5), where L represents the logarithm of the highest dilution, d is the difference in logarithmic dilution, and S is the sum of the proportion of positive (cytopathic effects were observed) wells [30,31]. The conversion from lgTCID50 to TCID50/mL is a standard antilog transformation: TCID50/mL = 10^(lgTCID50)^.

### 2.6. Construction of the Recombinant Vector

Total RNA was extracted from PEDV CHFJFQ-infected Vero cells (36 h post-infection) and reverse-transcribed into cDNA. A fragment or the full-length S gene of PEDV CHFJFQ was then amplified by PCR using the synthesized cDNA. The following primers were used to amplify a fragment of S gene: PEDV-S-QRT-F (CATTTCGCAAGAGCCGTTT) and PEDV-S-QRT-R (TTGTTGAATAGGCAGTTACGACC) and the full-length S gene: PEDV-S-F (CGGGATCCATGACGCCTTTAATTTACTTCTGG) and PEDV-S-R (GGAATTCTCAAGCGTAGTCTGGGACGTCGTATGGGTACTGCACGTGGACCTTTTCAA). The S gene fragment of PEDV CHFJFQ was ligated into pMD19™-T (6013; Takara, Dalian, China) following the manufacturer’s instructions, and the recombinant plasmid was designated p19T-S. The full-length S gene amplicon was then double-digested using BamHI (FD0054; Thermo Fisher) and EcoRI (FD0274; Thermo Fisher) and ligated into the pcDNA3.1+ (VT1001; Youbio, Changsha, China) vector using T4 DNA ligase (2011A; Takara). The resulting recombinant plasmid was designated pcDNA3.1-PEDV-S. The pcDNA3.1-TGEV-S plasmid, containing the TGEV S gene, was synthesized by Tsingke Biotech Co., Ltd. (Beijing, China).

### 2.7. Generation of PEDV CHFJFQ Pseudovirus

The pcDNA3.1-PEDV-S, pNL (Addgene, #18698, Cambridge, MA, USA), and psPAX2 (Addgene, #12260, USA) plasmids (8:6:6 μg) were co-transfected into 293T cells (100 mm culture dish, approximately 10 million cells). Transfection was performed using Lipofectamine™ 3000 (L3000-008; Invitrogen) according to the manufacturer’s instructions. After 12 h of transfection, the culture medium was replaced, and the supernatant was harvested every 24 h for three consecutive collections. The harvested pseudovirus was centrifuged at 3000 rpm for 10 min, and the supernatant was stored at −80 °C. Vero, ST, L929, 293A, IPEC-J2, and PK15 cells were then infected with the PEDV CHFJFQ pseudovirus. One milliliter of the pseudovirus was mixed with 2 × 10^6^ cells (2 mL) and incubated for 48 h. EGFP expression was detected by fluorescence microscopy to assess infection with the pseudovirus.

### 2.8. Cell Adsorption, Infection, and Proliferation Assays

The cellular adsorption characteristics of PEDV CHFJFQ were evaluated using six different cell lines (293A, Vero, L929, ST, IPEC-J2, and PK15). Prior to infection, each well of a 6-well plate was seeded with 1 × 10^6^ cells and incubated for 24 h to allow for cell attachment and growth. Subsequently, PEDV (MOI = 5) was added to the cells, and the plate was incubated at 4 °C for 2 h. After two washes with pre-cooled PBS, RNA was extracted using TRIzol™ (15596018, Thermo Fisher, Waltham, MA, USA). Viral copies were quantified by qPCR, using the recombinant vector p19T-S as a standard. A dilution series of p19T-S (1, 0.1, 0.01, 0.001, 0.0001, and 0.00001 ng) served as templates for generating the amplification curve and corresponding equation. The copy number of 1 ng of p19T-S was calculated as 6.02 × 10^23^ × (1 × 10^−^⁹/(2863 × 660)) ≈ 3.18 × 10^8^. For quantification, a standard curve was generated by plotting the log of the recombinant vector copy number (*y*-axis) versus the cycle threshold (Ct) value (*x*-axis). Viral copy numbers in experimental samples were then calculated based on the resulting regression equation.

To investigate the infection and replication characteristics of PEDV CHFJFQ, six cell lines (293A, Vero, L929, ST, IPEC-J2, and PK15) were infected with either PEDV CHFJFQ (MOI = 0.1) or a PEDV CHFJFQ pseudovirus. Following infection, cells were monitored by photography and harvested at 12, 24, 36, and 48 h post-infection (hpi). At each time point, protein was extracted for Western blot analysis, and RNA was extracted for reverse transcription quantitative PCR (RT-qPCR). In a parallel experiment, the infection was allowed to proceed for up to 60 hpi, with culture supernatants harvested every 12 h for further analysis. The viral titer was determined using the Spearman–Kärber method [30,31]. To verify successful infection with the PEDV CHFJFQ pseudovirus, 2 × 10^6^ cells were mixed with 1 mL of the pseudovirus (bringing the total volume to 2 mL) and incubated for 48 h. Subsequently, the expression of the enhanced green fluorescent protein (EGFP) reporter gene was evaluated using fluorescence microscopy. In addition, to assess the impact of trypsin on viral replication, Vero cells (5 × 10^5^) were infected with PEDV CHFJFQ (1 × 10^3^ TCID50) in 6-well plates with 2 mL of DMEM containing 2% FBS. After 2 h incubation at 37 °C, the media were removed and replaced with DMEM containing 10 μg/mL trypsin or with DMEM alone. Supernatants were collected every 12 h, and virus titers were determined using the Spearman–Kärber method [30,31].

### 2.9. Western Blotting and Reverse Transcription–Quantitative Polymerase Chain Reaction (RT-qPCR) Analysis

Cells were seeded in six-well plates at a density of 5 × 10^6^ cells per well and subsequently infected with PEDV strain CHFJFQ at a multiplicity of infection (MOI) of 0.1. Cells were harvested at 12, 24, 36, and 48 h post-infection (hpi). Proteins were extracted using RIPA buffer (P0013B, Beyotime, Shanghai, China), and their concentrations were determined using the Enhanced BCA Protein Assay Kit (Beyotime, P0010). Sixty micrograms of protein were loaded onto an SDS-polyacrylamide gel for electrophoresis, followed by transfer to a PVDF membrane. The membrane was washed twice with TBST and blocked with 5% skimmed milk at room temperature for 2 h. The PVDF membrane was then incubated with the primary antibody at 4 °C for 8 h. The primary antibodies, anti-PEDV NP (SD-2-1, Medgene Labs, Brookings, SD, USA) and GAPDH, were diluted 1:1000 in 5% skimmed milk. The PVDF membrane was washed twice with TBST and incubated with IRDye^®^ 800 CW donkey anti-mouse IgG secondary antibody for 2 h at room temperature. The secondary antibody was diluted 1:20,000 in 5% skimmed milk. After two additional washes with TBST, images were visualized using an LI-COR Odyssey infrared fluorescence scanner (LI-COR).

For RT-qPCR, one microgram of RNA was reverse-transcribed into cDNA and diluted to 50 ng/μL. RT-qPCR was then performed using 100 ng of cDNA. To detect the PEDV S gene and measure the expression levels of the N gene, RT-qPCR was carried out using ChamQ Universal SYBR qPCR Master Mix (Q711-02/03, Vazyme) with a QuantStudio 6 Flex system (4485697, Thermo Fisher). The primers are listed in Supplementary Table S2. The reactions were incubated at 95 °C for 30 s, followed by 40 cycles of 95 °C for 10 s and 60 °C for 30 s. Gene expression levels were calculated using the 2^−ΔΔCt^ method.

### 2.10. Immunofluorescence Assay

To visualize PEDV CHFJFQ proliferation in cells, we assessed the expression of the viral nucleocapsid (N) protein via immunofluorescence. Briefly, 1 × 10^5^ cells were cultured in confocal dishes and infected with PEDV CHFJFQ (MOI = 0.1). At 48 h post-infection (hpi), cells were fixed with Immunostaining Fix Solution (P0098-100 mL; Beyotime) and permeabilized with Immunostaining Permeabilization Buffer containing Triton X-100 (P0096-100 mL; Beyotime), followed by blocking with QuickBlock™ Blocking Buffer (P0260; Beyotime). The primary antibody, anti-PEDV NP, was diluted 1:400 in Immunol Staining Primary Antibody Dilution Buffer, and the secondary antibody, Alexa Fluor™ 546 donkey anti-mouse IgG (H + L), was diluted 1:1000 in Immunol Fluorescence Staining Secondary Antibody Dilution Buffer. All experiments were conducted according to the respective manufacturer’s instructions. Imaging was performed using an LSM 780 laser confocal system (Zeiss, Oberkochen, Germany).

### 2.11. Animal Experimental Challenge and Ethical Statement

Fifteen colostrum-deprived newborn piglets, tested negative for porcine acute diarrhea syndrome coronavirus (SADS), PEDV, transmissible gastroenteritis virus (TGEV), porcine delta coronavirus, porcine rotavirus, porcine reproductive and respiratory syndrome virus (PRRSV), pseudorabies virus (PRV), and porcine circovirus (PCV1 and PCV2), were purchased from a pig farm (Lianjiang Hongyun Farming and Animal Husbandry Co., Ltd., Lianjiang, Fujian, China) for challenge studies. The piglets were randomly assigned to three groups (*n* = 5 per group): a control group, an oral inoculation PEDV CHFJFQ group, and a neck muscle inoculation PEDV CHFJFQ group. The control group was inoculated with 1 mL DMEM via both oral and intramuscular injection. The oral and intramuscular inoculation groups were each administered 5 × 10^6^ TCID50 (1 mL) of PEDV CHFJFQ. The piglets were fed milk, and their feeding and health statuses were monitored every 3 h. Cases of diarrhea were recorded. The experiment was terminated when piglets were unable to stand or eat, at which point the affected piglets were euthanized using carbon dioxide (CO_2_). The carcasses were examined for macroscopic and microscopic lesions, and the expression of the PEDV CHFJFQ S gene was assessed in the heart, liver, spleen, lung, kidney, brain, and intestine using RT-qPCR. Additionally, the expression of PEDV N antigen in the intestine, lung, and spleen of all the experimental animals was evaluated by immunohistochemistry.

Thirty male SPF C57BL/6 mice (4 weeks old) were purchased from Shanghai SLAC Laboratory Animal Co., Ltd. (Shanghai, China). Ten mice were randomly assigned to either the control group or the PEDV CHFJFQ inoculation group. The control group was orally inoculated with 0.1 mL DMEM, while the PEDV inoculation group received 5 × 10^5^ TCID50 of PEDV CHFJFQ (0.1 mL) orally. The mice were monitored for diarrhea every 6 h and euthanized with CO_2_ at 72 h post-infection (hpi). The expression of the PEDV N gene in the heart, liver, spleen, lung, kidney, brain, and small intestine was evaluated by RT-qPCR and immunohistochemistry.

Twenty additional mice were randomly assigned to either the control or PEDV inoculation group. Their survival status was monitored every 24 h for 14 days, and a survival curve was plotted. Mice were euthanized with CO_2_ at 15 days post-infection (dpi). The PEDV infection group was orally inoculated with 5 × 10^5^ TCID50 of PEDV CHFJFQ (0.1 mL), and the control group received the same volume of DMEM. The protocol for the animal experiments was approved by the Animal Care and Use Committee of Fujian Normal University (approval number: IACUC-20220039).

### 2.12. Hematoxylin and Eosin (H&E) Staining and Immunohistochemistry

Tissues were fixed in 4% paraformaldehyde for 12 h at room temperature. The samples were dehydrated for 30 min using graded ethanol concentrations (30%, 50%, 70%, 90%, and 100%) and then cleared with a 1:1 mixture of ethanol and xylene. The tissues were subsequently treated overnight with a 1:1 mixture of paraffin and xylene, and xylene was used to replace the alcohol in the tissue blocks. The tissues were sectioned into 5 μm slices, stained with hematoxylin and eosin (H&E), and subjected to immunohistochemistry (PEDV-N antibody was diluted 1:400).

### 2.13. Bioinformatics

MUSCLE software (v.5.1.linux64) [32], which performs fast multiple sequence alignment with high accuracy, was used to align 247 full-length genome sequences (245 PEDV genomes and 2 bat coronavirus genomes). The maximum number of iterations was set to 2. IQtree software (v.2.2.2.3) was used to construct rootless phylogenetic trees using a maximum likelihood algorithm [33]. ModelFinder was employed to evaluate various substitution models and select the most appropriate model for the sequence set to construct the evolutionary tree. The phylogenetic tree data were further analyzed using iTOL (https://itol.embl.de/). The Recombination Detection Program version 5 (RDP5) was used to analyze possible recombination events in the PEDV CHFJFQ strain [34,35].

Phylogenetic analysis of the S genes from 9 bat coronaviruses and 2 PEDV strains (PEDV CHFJFQ and PEDV CV777) was performed using MEGA software (v.7.0.26) [36]. Sequence alignment of the 11 groups was conducted using MUSCLE in MEGA to obtain alignment results for the 11 S protein groups, and a phylogenetic tree was constructed using the maximum likelihood method. BLAST (v.2.16.0) was used to compare different positions of genes across multiple groups to calculate similarity values for each gene, and a heatmap was generated using the R (v.4.1.2) package pheatmap (v.1.0.12).

### 2.14. Statistical Analysis and Image Processing

Images of Western blots, H&E staining, transmission electron microscopy, cells, immunohistochemistry, and immunofluorescence were processed using Adobe Illustrator 2022 software, and sequence data were analyzed using BioEdit software (v.7.2.5) [37,38]. Mantel–Cox test or unpaired *t*-tests (GraphPad Prism 5.0, GraphPad Software, San Diego, CA, USA) were used to assess differences between groups. The results are presented as means ± standard error of the mean for each treatment, and *p* < 0.05 was considered a significant difference.

## 3. Results

### 3.1. Genomic Characteristics and Phylogenetic Analysis of the PEDV CHFJFQ

The genome of PEDV CHFJFQ consists of 27,953 nucleotides (excluding poly-A tails), as determined by sequencing. Compared to the classical PEDV strain CV777 (GenBank accession AF353511.1), the genome of PEDV CHFJFQ exhibits several nucleotide substitutions, deletions, and insertions, including a 1-nucleotide insertion and a 5-nucleotide deletion in the 5′ untranslated region (Figure 1b), a 24-nucleotide deletion in ORF1a/1b (Figure 1c), a 3-nucleotide deletion in the S gene (Figure 1d), and a 49-nucleotide deletion in ORF3 (Figure 1e). These nucleotide variations result in corresponding amino acid changes. There were 56 amino acid substitutions and 1 amino acid deletion (154Arg) in the S protein (Supplementary Figure S2a). In ORF3, there were 10 amino acid insertions (frameshift mutations), 3 amino acid substitutions (21Val to Ala, 54Val to Ile, and 79Val to Ile), and 143 amino acid deletions (a frameshift mutation leading to premature translation termination) (Supplementary Figure S2b). In the E protein, two amino acid substitutions were observed (11Val to Ala and 76Val to Ile) (Supplementary Figure S2c). Additionally, four and nine amino acid substitutions were identified in the M and N proteins, respectively (Supplementary Figure S2d,e). Unlike the attenuated DR13 (GenBank accession JQ023162.1), which shows a 21-nucleotide deletion in the E gene compared to the CV777 strain, the CHFJFQ strain did not exhibit this deletion (Figure 1f). In the M and N genes, no nucleotide deletions or insertions were observed in any of the strains (CHFJFQ, AJ1102 (GenBank accession JX188454.1), virulent DR13 (GenBank accession JQ023161.1), and attenuated DR13 (GenBank accession JQ023162.1)) when compared to CV777 (Figure 1g,h).

**Figure 2:**
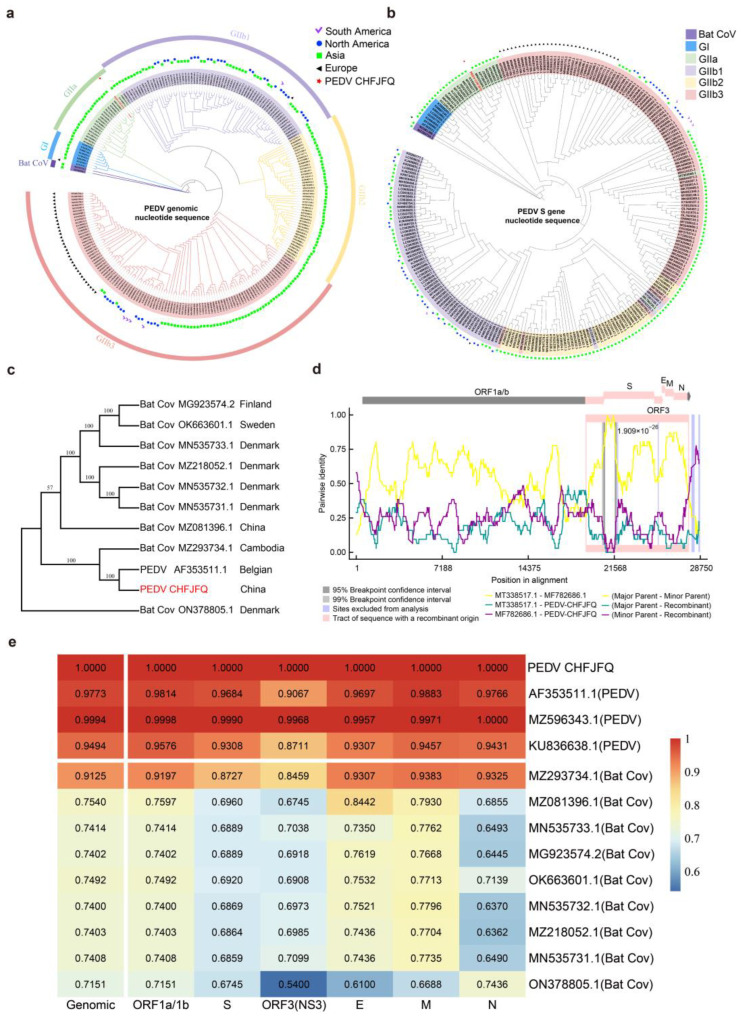
Phylogenetic analysis, nucleotide similarity, and recombination analysis of PEDV CHFJFQ. To explore the evolution and recombination of PEDV, 245 complete PEDV genome sequences and 2 bat coronavirus genome sequences were analyzed using MUSCLE, IQtree, MEGA7, and RDP5. PEDV CHFJFQ, marked with red stars, belongs to the GIIa subgroup. (**a**) The evolution and global distribution of PEDV were analyzed based on genome-wide data. (**b**) The evolution and global distribution of PEDV were also analyzed based on the S gene. (**c**) Phylogenetic analyses of PEDVs and bat coronaviruses. (**d**) PEDV CHFJFQ is potentially a recombinant strain, with CH/HNXX/2016 as the major parent and NW17 as the minor parent. The recombination regions are highlighted in pink. (**e**) Nucleotide similarity analyses of PEDV and bat coronavirus genomes. MUSCLE software was used to align 247 full-length genome sequences, including 245 PEDV and 2 bat coronavirus genomes. The following command was executed: muscle -in seqs.fa -out seqs.afa -maxiters 2, with a maximum iteration limit of 2. Subsequently, IQtree software was used to construct rootless phylogenetic trees based on a maximum likelihood algorithm, executed with the command iqtree -s seqs.afa -nt 50 -iteration 2. ModelFinder was used to assess various nucleotide substitution models and select the most suitable one for constructing the evolutionary tree. The phylogenetic tree data were further analyzed using the interactive Tree Of Life (iTOL) platform (https://itol.embl.de/, accessed on 12 February 2025). Additionally, Recombination Detection Program version 5 (RDP5) was used to identify potential recombination events within the PEDV CHFJFQ strain. For the phylogenetic tree analysis of the S genes of 9 bat coronaviruses and 2 PEDV strains (PEDV CHFJFQ and PEDV CV777), MEGA software was used. Sequence alignment across 11 groups was performed using MUSCLE integrated within MEGA, producing 11 sets of aligned S protein sequences. A phylogenetic tree was then constructed using a maximum likelihood approach. BLAST was applied to compare different gene positions across multiple groups, providing similarity values for each gene. The resulting data were visualized as a heatmap using the R package pheatmap.

The genome and S gene sequences of 245 PEDV strains from 20 countries, along with 2 bat coronavirus strains, were analyzed, and two phylogenetic trees were constructed (Figure 2a,b). PEDVs can be classified into two genogroups, GI and GII. GII is further divided into the GIIa and GIIb subgroups, with GIIb being subdivided into the GIIb1, GIIb2, and GIIb3 clades. The PEDV CHFJFQ strain belongs to the GIIa subgroup (Figure 2a,b).

The distribution of PEDV genogroups showed regional variations. Asia encompassed all PEDV subgroups, while the epidemic strains in South and North America predominantly belonged to the GIIb1 and GIIb3 subgroups. In Europe, the predominant subgroup for the epidemic strains was GIIb3 (Figure 2a,b). PEDVs share a common ancestor with bat coronaviruses (Figure 2a,c). The data also suggest that PEDV CHFJFQ may be a recombinant strain, with CH/HNXX/2016 as the major parent and NW17 as the minor parent (Figure 2d). The recombination region spans from the 3′ end of ORF1a/b to the end of the N gene (Figure 2d). PEDV CHFJFQ shows 91.25% genomic nucleotide similarity and 95.4% amino acid similarity with a bat coronavirus (PREDICT/GCS-011, GenBank accession MZ293734.1) isolated from Cambodia. The nucleotide similarities between these two viruses in various genes were as follows: ORF1a/1b (91.97%), S (87.27%), ORF3 (84.59%), E (93.07%), M (93.83%), and N (93.25%) (Figure 2e, Table 1). Therefore, PEDVs and the bat coronavirus PREDICT/GCS-011 can be considered conspecific. Additionally, the nucleotide similarity between the bat coronaviruses analyzed and PEDV CHFJFQ ranged from 71.51% to 91.25% (Figure 2e). These bat coronaviruses were primarily distributed in Switzerland, Finland, Denmark, Cambodia, and China (Figure 2c). Among PEDV strains, PEDV CHFJFQ showed the highest nucleotide similarity (99.94%) with the Chinese isolate SD 2019 (GenBank accession MZ596343.1) (Figure 2e) and the lowest similarity (94.94%) with the British isolate YorkS-2000 (GenBank accession KU836638.1) (Figure 2e). The genomic nucleotide similarity between PEDV CHFJFQ and PEDV CV777 (GenBank accession AF353511.1) was 97.73%, with nucleotide similarities in ORF1a/1b, S, ORF3, E, M, and N between these two strains being 98.14%, 96.84%, 90.67%, 96.97%, 98.83%, and 97.66%, respectively (Figure 2e).

### 3.2. Biological Characteristics of the PEDV CHFJFQ Strain in Vitro

**Figure 3:**
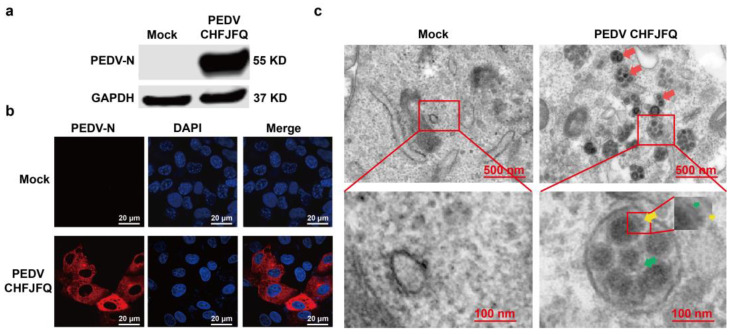
The expression of PEDV N protein and virus particles in Vero cells. (**a**) The expression of PEDV N protein in Vero cells was detected by Western blotting at 48 hpi. Vero cells (5 million per well) in six-well plates were co-cultured with PEDV CHFJFQ (MOI: 0.1). Cells were harvested at 48 hpi, and total protein was extracted for Western blotting. Mouse monoclonal anti-PEDV N protein antibody was used to detect the expression of PEDV N protein, with GAPDH as the control. (**b**) The expression of PEDV N protein in Vero cells was detected by immunofluorescence at 48 hpi. Vero cells (1 × 10^5^) were co-cultured with PEDV CHFJFQ (MOI = 0.1) in confocal dishes. Cells were fixed, permeabilized, and probed with mouse monoclonal anti-PEDV N protein antibody, followed by Alexa Fluor™ 546 Donkey anti-mouse IgG (H + L) (red) for confocal immunofluorescence detection of PEDV N protein expression. Nuclei were stained with DAPI (blue). (**c**) Morphology of PEDV virions observed by transmission electron microscopy (48 hpi). Vero cells (5 × 10^5^ per well) were co-cultured with PEDV CHFJFQ (MOI: 0.01) in six-well plates. Cells were fixed at 48 hpi for subsequent TEM examination. Virus particles (red arrow): 80–100 nm; envelope (green arrow): 5–7 nm; spike (yellow arrow): 16–20 nm. The experiment was independently repeated three times, and the presented figure depicts the results of one representative replicate. Full-length blots are presented in Supplementary Figure S4.

To further investigate the infection characteristics of the PEDV CHFJFQ strain in vitro, we examined its infectivity across various cell types by evaluating its ability to bind, infect, and replicate within cells. After seven passages (P7), PEDV CHFJFQ exhibited robust proliferation in Vero cells, with PEDV N protein expression detected by Western blotting and immunofluorescence at 48 hpi (Figure 3a,b). Transmission electron microscopy revealed mature PEDV particles in Vero cells, with a particle size of approximately 80–100 nm and a coronal spike of about 20 nm on the particle surface (Figure 3c). The proliferation of PEDV CHFJFQ in Vero cells was independent of trypsin treatment, with a titer of 1.3 × 10^7^ TCID50/mL (Supplementary Figure S3).

**Figure 4:**
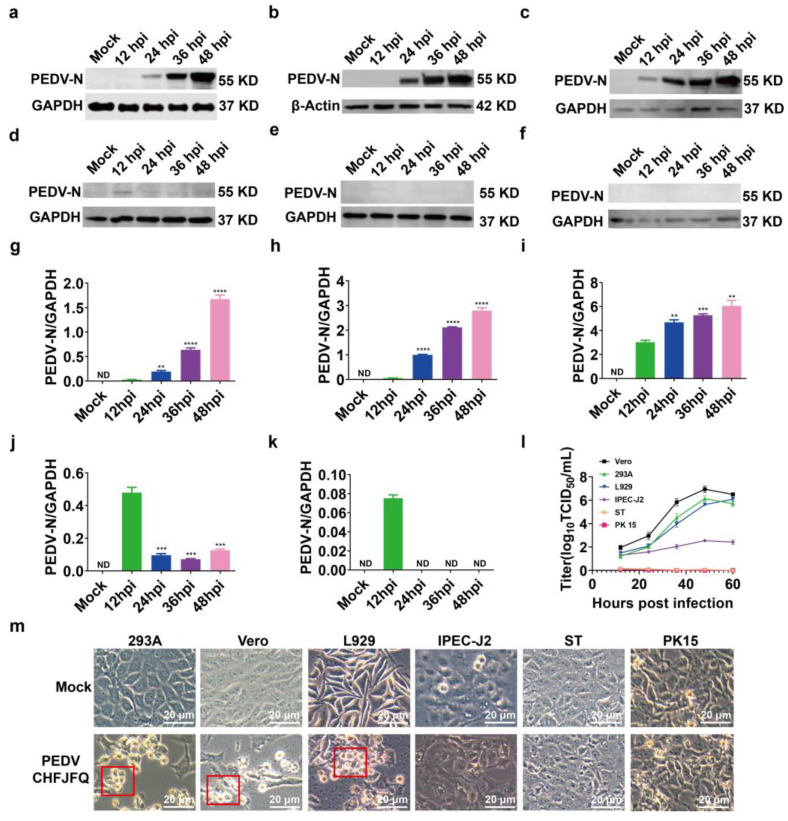
Proliferative characteristics of PEDV CHFJFQ in vitro. (**a**–**f**) The expression of PEDV N protein in 293A, Vero, L929, IPEC-J2, ST, and PK15 cells was detected by Western blotting at various time points post-infection (*n* = 3). Six cell lines (5 × 10^5^ cells per well) were co-cultured with PEDV CHFJFQ (MOI = 0.1) in 6-well plates. Cells were harvested at 12, 24, 36, and 48 h post-infection (hpi), and total protein was extracted for Western blot analysis. Anti-PEDV N protein antibody was used to detect PEDV N protein expression, and GAPDH or β-actin was used as endogenous controls. (**g**–**k**) The relative expression of PEDV N protein in relation to GAPDH or β-actin from (**a**–**e**) was analyzed using ImageJ (v.1.8.0) (*n* = 3). (**l**) Viral titers at different time points of PEDV infection in 293A, Vero, L929, IPEC-J2, ST, and PK15 cells. Six cell lines (5 × 10^5^ cells per well) were co-cultured with PEDV CHFJFQ (MOI = 0.1) in 6-well plates. The culture supernatants were harvested at 12, 24, 36, and 48 hpi, and the virus titers were determined using the Spearman–Kärber method (*n* = 3). (**m**) Cytopathic effects were observed by microscopy and photographed at 48 hpi of PEDV CHFJFQ infection. The red box highlights the cytopathic effects caused by PEDV CHFJFQ infection. Unpaired *t*-tests (GraphPad Prism 5.0, GraphPad Software, San Diego, CA, USA) were used to test differences between groups. Data are presented as means ± standard error of the mean for each treatment. ** *p* < 0.01, *** *p* < 0.001, and **** *p* < 0.0001 vs. control group (12 hpi); ND: none detected. Sample sizes are indicated in brackets. The full-length blots are presented in Supplementary Figure S7.

In addition to porcine cells, PEDV CHFJFQ also exhibits sensitivity to human and mouse cells. In 293A, Vero, and L929 cells, both the level of PEDV N protein and the viral titer increased with prolonged infection time (Figure 4a–l). The relative expression of the PEDV S gene also increased with the duration of infection (Supplementary Figure S5). The cytopathic effects of PEDV CHFJFQ were characterized by cell shrinkage and eventual cell detachment (highlighted in the red box in Figure 4m), with no observed cell fusion or syncytium formation (Figure 4m). However, PEDV CHFJFQ was unable to proliferate in porcine ST and PK15 cell lines (Figure 4e,f,k) but was detected at a lower level in IPEC-J2 cells, without causing noticeable cytopathic effects (Figure 4d,j,m). Although weak banding was observed in ST cells infected with PEDV at 12 hpi, this likely represents the early expression of PEDV proteins following initial viral entry, but not productive viral replication. This interpretation is consistent with our finding that PEDV CHFJFQ is unable to proliferate in ST cells, meaning it cannot complete a full replication cycle and produce infectious progeny virus.

Interestingly, PEDV CHFJFQ was able to bind to all Vero, 293A, L929, ST, IPEC-J2, and PK15 cells (Supplementary Figure S6). These findings suggest the presence of PEDV receptors or co-receptors on the surface of these cells. The coronavirus infection process is a sequential cycle consisting of adsorption, internalization, genome replication, virion assembly, and release. The interruption of any stage in this cycle will result in abortive infection. As shown in Figure 4e–f and Supplementary Figure S5e–f, PEDV CHFJFQ is unable to proliferate in ST and PK15 cells. However, the specific step in the infection process that is impaired remains unclear.

**Figure 5:**
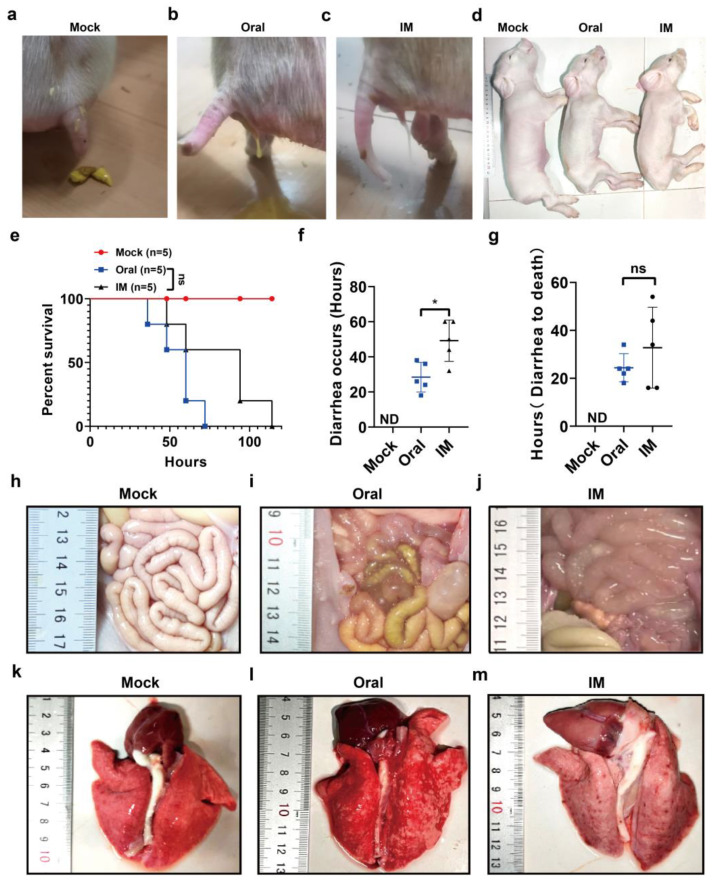
Clinical symptoms and survival of piglets challenged with PEDV CHFJFQ. (**a**–**c**) Both oral and intramuscular inoculation of PEDV CHFJFQ resulted in diarrhea. (**d**) Dehydration was observed in piglets following both oral and intramuscular inoculation of PEDV CHFJFQ (60 hpi). (**e**) Survival curves of piglets challenged with PEDV CHFJFQ (*n* = 5). (**f**) Time to onset of diarrhea following inoculation with PEDV CHFJFQ (*n* = 5). (**g**) Time to death in piglets due to diarrhea (*n* = 5). (**h**–**j**) Oral or intramuscular inoculation of PEDV CHFJFQ caused intestinal thinning in piglets. (**k**–**m**) Pulmonary hemorrhage was observed in piglets following oral or intramuscular inoculation with PEDV CHFJFQ. The control group was inoculated with 1 mL of DMEM via oral and intramuscular injection, respectively. The oral and intramuscular inoculation groups received 1 mL of 5 × 10^6^ TCID50 PEDV CHFJFQ. The health status of the piglets was monitored every 3 h, and the experiment was terminated when piglets were unable to stand or eat. Data were analyzed using the Mantel–Cox test (**e**), and unpaired *t*-tests (**f**,**g**) (GraphPad Prism 5.0, GraphPad Software, San Diego, CA, USA) were used to test differences between groups. Data are presented as means ± standard error of the mean for each treatment. * *p* < 0.05 vs. control group (oral), ns: *p* > 0.05 vs. control group (oral), ND: none detected. Sample sizes are indicated in brackets.

The addition of data from Figure 5c demonstrates that both ST and PK15 cells are capable of adsorbing PEDV CHFJFQ. Therefore, the failure of PEDV CHFJFQ to establish a productive infection in ST and PK15 cells is likely due to a block in internalization, genome replication, virion assembly, or release. The use of a pseudovirus system allowed us to specifically focus on the viral entry process. By using a heterologous viral backbone (VSV) expressing the PEDV spike protein, we could isolate the effects of the spike protein on cell entry from other viral factors that might be influencing the outcome in infections with the complete, replicating virus. This allows for a more targeted investigation of receptor binding and membrane fusion. Furthermore, the pseudovirus system enabled us to easily incorporate reporter genes (GFP) to quantitatively measure viral entry efficiency. Given that infection with pseudovirus typically involves cell binding and entry, we further investigated the ability of the PEDV CHFJFQ pseudovirus to infect cells. PEDV CHFJFQ successfully infected ST cells but not PK15 cells (Supplementary Figure S6d), indicating that different mechanisms may be involved in PEDV CHFJFQ infection or proliferation in these cell lines, possibly due to the loss of host-dependent factors (such as mutations, silencing, or low expression of specific genes).

### 3.3. Pathogenicity of the PEDV CHFJFQ Strain in Piglets

**Figure 6:**
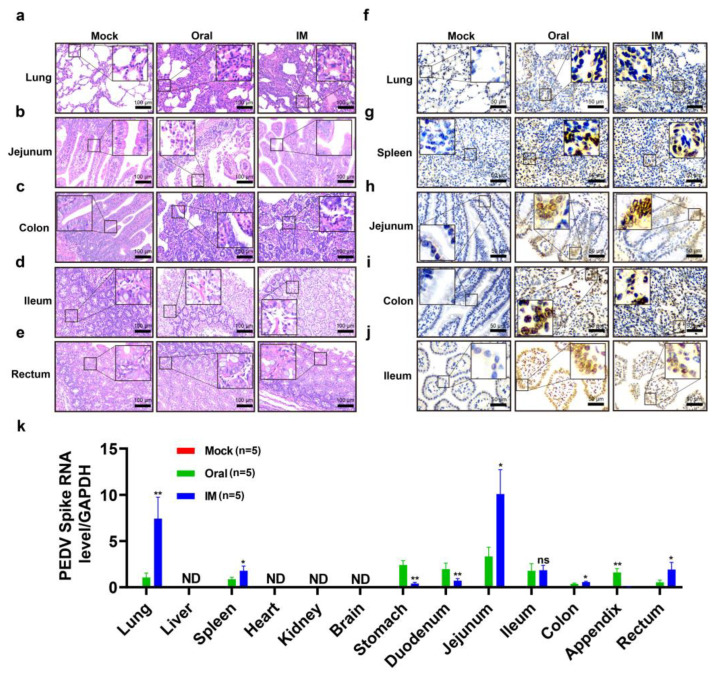
Pathological changes induced by PEDV CHFJFQ and its distribution in piglets. (**a**) PEDV CHFJFQ causes thickening of the alveolar walls and pulmonary inflammation in piglets. (**b**) PEDV CHFJFQ induces villous atrophy and epithelial cell shedding in the jejunum of piglets. (**c**) The colon of a piglet inoculated with PEDV CHFJFQ shows diffuse atrophic enteritis. (**d**) PEDV CHFJFQ leads to the shedding of villous epithelial cells in the ileum of piglets. (**e**) Intramuscular inoculation of PEDV CHFJFQ induces shedding of rectal villus epithelial cells in piglets. (**f**–**j**) Immunohistochemical analysis of PEDV N protein expression in the lung, spleen, jejunum, ileum, and colon. (**k**) The relative content of the PEDV CHFJFQ S gene in each organ was detected by RT-qPCR (*n* = 5). ND: none detected. Unpaired *t*-tests (GraphPad Prism 5.0, GraphPad Software, San Diego, CA, USA) were used to test differences between groups. Data are presented as means ± standard error of the mean for each treatment. * *p* < 0.05, ** *p* < 0.01 vs. control group (oral), ns: *p* > 0.05 vs. control group (oral), ND: none detected. Sample sizes are indicated in brackets.

Although typically associated with enteric disease, characterized by diarrhea, vomiting, and dehydration leading to mortality in piglets, PEDV has also been found to exhibit systemic spread to tissues beyond the intestine [39]. Oral or intramuscular inoculation resulted in diarrhea, vomiting, and death in piglets (Figure 5a–e). Notably, oral inoculation caused diarrhea earlier than intramuscular injection (Figure 5f). The visible damage caused by PEDV CHFJFQ in piglets also included pulmonary hemorrhage (Figure 5i,j,l,m). H&E staining revealed that PEDV CHFJFQ infection caused lung inflammation, intestinal villus atrophy, epithelial cell shedding, and liver injury, while no significant effects were observed in the brain, heart, spleen, or kidneys (Figure 6a–e, Supplementary Figure S8). Immunohistochemistry and RT-qPCR demonstrated that PEDV replication occurred in the lung, spleen, jejunum, colon, and ileum but not in the liver (Figure 6f–k). Based on the observed PEDV proliferation in these tissues, we conclude that PEDVs can cause direct damage to the intestine and lung and indirect damage to the liver. These findings suggest that, unlike SARS-CoV, SARS-CoV-2, and MERS-CoV infections, which primarily cause respiratory symptoms such as respiratory distress, cough, and pneumonia, PEDV not only causes intestinal damage but also induces lung injury, which may contribute to mortality in piglets.

### 3.4. Pathogenicity of the PEDV CHFJFQ Strain in Mice

**Figure 7:**
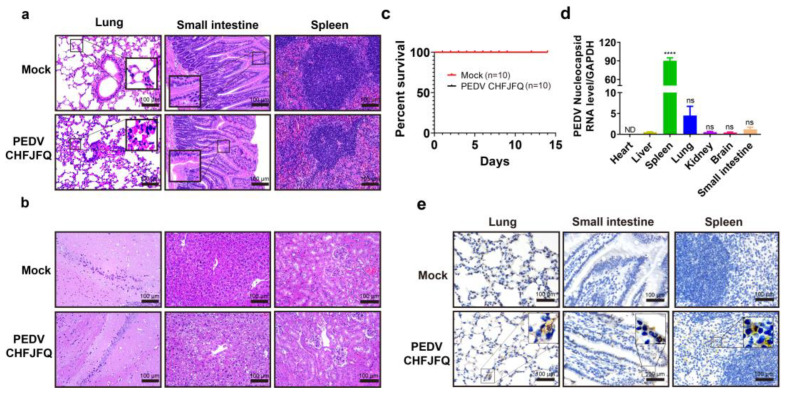
Clinical symptoms, survival curves, and pathological changes in mice challenged with PEDV CHFJFQ. (**a**,**b**) PEDV CHFJFQ caused lung and liver injury as well as intestinal villus atrophy in mice. Hematoxylin–eosin (H&E) staining was used to observe the pathological changes in mice induced by PEDV CHFJFQ infection at 72 hpi (*n* = 5). (**c**) Survival curves of mice challenged with PEDV CHFJFQ (*n* = 10). (**d**) The relative content of the PEDV CHFJFQ N gene in each organ was detected by RT-qPCR (*n* = 5). (**e**) Immunohistochemical detection of PEDV N protein expression in the lung, jejunum, and spleen at 72 hpi (*n* = 5). Mice in the PEDV infection group were orally inoculated with 5 × 10^5^ TCID50 of PEDV CHFJFQ (0.1 mL), while the control group received the same volume of DMEM. Unpaired *t*-tests (GraphPad Prism 5.0, GraphPad Software, San Diego, CA, USA) were used to test differences between groups. Data are presented as means ± standard error of the mean for each treatment. **** *p* < 0.0001 vs. control group (liver), ns: *p* > 0.05 vs. control group (liver), ND: none detected. Sample sizes are indicated in brackets.

A variety of coronaviruses have demonstrated the ability for cross-species transmission [40], but whether PEDVs have this ability remains unclear. Given its sensitivity to mouse cells and the potential for exposure to mice during pig feeding, we assessed the pathogenicity and infection characteristics of PEDV CHFJFQ in mice. Inoculation with PEDV CHFJFQ did not cause diarrhea or death in the mice, but H&E staining revealed pulmonary inflammation, thickened alveolar walls, and intestinal villus atrophy (Figure 7a–c). Additionally, RT-qPCR and immunohistochemistry showed that PEDV CHFJFQ was present in the lung, intestine, and spleen of the mice (Figure 7d–e).

## 4. Discussion

PEDV infection has emerged as a serious obstacle hindering the growth and advancement of the pig breeding industry. Since 2010, highly mutated strains of PEDV have emerged in China, the United States, Canada, and Europe, resulting in significant economic losses to the global pork industry [23,41,42]. Currently available commercial vaccines for porcine epidemic diarrhea virus (PEDV) prevention, such as inactivated vaccines based on the CV777 strain and live-attenuated vaccines derived from the AJ1102 strain, have demonstrated limited efficacy [43]. Despite these efforts, PED outbreaks continue to occur frequently in China, highlighting the urgent need for more effective vaccines to prevent PEDV infection. A thorough understanding of PEDV evolution, infection characteristics, and host range is vital for designing and implementing effective PEDV control measures.

Numerous novel PEDV strains have been isolated and identified, and these strains can be classified into GI, GII, or GIII genogroups based on genomic sequences or the S1 or ORF3 gene sequences, which can be further subdivided into subgroups [44]. Consistent with previous studies, our genome-wide evolutionary analysis of 245 PEDV strains from around the world confirmed the division of PEDV into GI and GII genogroups. The GII group was further subdivided into GIIa and GIIb subgroups, with the GIIb subgroup further classified into GIIb1, GIIb2, and GIIb3 clades. As previously reported, the majority of PEDV isolates belonged to the GII group [23]. However, our results indicate that previously identified recombinant and S-indel-like groups fall within the GIIb3 clade [23,45]. Notably, the GIIb3 clade exhibits a broad geographical distribution, encompassing South America, North America, Asia, and Europe. Furthermore, PEDV evolution has been particularly rapid in China [41]. We identified PEDV CHFJFQ as a potential recombinant strain, with CH/HNXX/2016 as the major parent and NW17 as the minor parent. While the specific mechanisms underlying this recombination remain to be elucidated, it is plausible that inter-provincial trade contributed to the co-localization and subsequent recombination of these genetically distinct PEDV strains [43]. In general, strains within the same group tend to share similar virulence. Both PEDV HLJBY (GenBank accession KP403802.1) [46] and PEDV CHFJFQ belong to the GIIa clade. However, unlike PEDV CHFJFQ, PEDV HLJBY was not lethal to piglets, suggesting that strains within the same group may exhibit differences in virulence [46]. Similarly, the attenuated PEDV strain DR13 (GenBank accession JQ023162.1), which also belongs to the GIIa subgroup, was non-lethal to piglets [10]. Notably, unlike attenuated DR13, PEDV CHFJFQ does not exhibit a 21-nucleotide deletion in the E gene. This suggests that the integrity of the E gene plays a crucial role in maintaining PEDV virulence.

The S protein is the key viral protein that stimulates the production of PEDV-neutralizing antibodies [47]. A significant challenge is that the S protein in GII strains (CHFJFQ, GenBank accession OP688373.1, and AJ1102, GenBank accession JX188454.1) is highly divergent from that of GI strains (represented by CV777, GenBank accession AF353511.1), bearing multiple mutations. This divergence may explain the limited protection provided by traditional vaccines developed against GI strains to protect against GII strains. The CHFJFQ strain was isolated from pig farms using standardized PEDV vaccines, suggesting a potential for vaccine escape. However, further studies are needed to determine whether specific mutations in CHFJFQ alter its antigenicity or neutralization susceptibility. Our observation of distinct PEDV genotype distributions further complicates vaccine design: Asia exhibits a wide array of genotypes, while South and North America primarily feature GIIb1 and GIIb3, and Europe is mainly represented by GIIb3. The existence of multiple genotypes may also explain the poor efficacy of the current PEDV vaccine, as most commercial PEDV vaccines are based on a single genotype [48]. In experiments assessing the cross-protective potential of G2a and G2b strains, piglet immunization and challenge studies revealed limited cross-protection between these genotypes [49]. To overcome this, researchers purified full-length S protein trimers from both G2a and G2b and developed a bivalent subunit vaccine targeting PEDV G2a/G2b S proteins (G2a/G2b-S). The subsequent results demonstrated that the G2a/G2b-S bivalent subunit vaccine conferred protection against both G2a and G2b strains [49]. Therefore, we propose that multi-genotype vaccines be developed for use in Asia, while single-genotype vaccines may be sufficient for Europe and North America.

Porcine epidemic diarrhea virus (PEDV) infection via the nasal route can elicit intestinal symptoms in piglets, with dendritic cells and CD3^+^ T lymphocytes implicated in the trafficking of the virus from the nasal cavity to the intestinal tract [50]. Similarly, intramuscular infection with PEDV CHFJFQ can also lead to intestinal symptoms in piglets. Thus, we propose that dendritic cells and CD3^+^ T cells may play a critical role in the transport of PEDV CHFJFQ from muscle to the intestine, although further experimental evidence is required. The role of PEDV infection in the lungs remains controversial. While some studies have indicated that nasal inoculation of PEDV only infects the upper respiratory tract and not the lungs, others have shown that PEDV can infect the lungs, liver, kidneys, spleen, and other organs [51,52]. Consistent with these findings, we observed that PEDV CHFJFQ induces lung injury in piglets via both oral and intramuscular routes, with PEDV antigen detectable in the lungs. This pulmonary infection may contribute to the mortality observed in piglets. Additionally, we found that PEDV CHFJFQ proliferates in the spleen, potentially representing a critical mechanism for immune evasion. Given that the spleen is a key immune organ vital for B cell development and maturation [53], PEDV infection in this organ may impair B cell maturation, ultimately leading to immunosuppression.

Studies have shown that specific mutations (A605E, E633Q, and R891G) within the spike protein of the Vero cell-adapted PEDV strain DR13^att^ are critical for its efficient replication in Vero cells, as evidenced by comparison to the highly virulent parental strain DR13^par^ [54]. Paralleling this observation, PEDV CHFJFQ, also a Vero cell-adapted strain, possesses these same mutations within its spike protein relative to the CV777 strain. To further characterize the cellular tropism of PEDV CHFJFQ, we assessed its ability to replicate in various cell lines. The results revealed that this strain efficiently proliferated and induced cytopathic effects in 293A, L929, and Vero cells, confirming its status as a multi-cell-adapted strain. The efficient infection of L929 cells by PEDV CHFJFQ was an unexpected finding, particularly given that L929 cells are a well-established model for studying innate immune signaling pathways [55]. Furthermore, given reports of PEDV infection in bat cells (TV1-LU) [28] and our observation of L929 cell infection, we propose that mice and bats may represent potential hosts for PEDV.

Previous studies have implicated the S and ORF3 proteins as key determinants of PEDV cellular adaptation [1,54]. Cell-adapted PEDV strains are often characterized by large nucleotide deletions in the ORF3 gene [56]. Consistent with this, the PEDV CHFJFQ strain exhibits a 49 nt deletion in ORF3, resulting in a truncated protein of 91 amino acids compared to the 224 amino acids encoded by the classical strain CV777. Given the known interaction and co-localization of the S protein with ORF3 in the cell membrane or cytoplasm [56], this interaction may play a role in PEDV cellular adaptation and virulence. While the overexpression of ORF3 has been shown to inhibit PEDV rescue in vitro, other studies suggest that full-length ORF3 enhances virulence and replication of recombinant PEDV strains in vitro [4,57,58]. Therefore, further investigation into the interplay between ORF3 and the S protein, and its impact on PEDV proliferation and virulence, is warranted.

Coronaviruses such as SARS-CoV-2 and MERS-CoV are believed to have originated from bats [59,60], a hypothesis also proposed for PEDV [28]. Notably, we found that PEDV CHFJFQ shared evolutionary ancestry with a bat coronavirus (PREDICT/GCS-011, GenBank accession MZ293734.1) from Cambodia. Because mouse cells are sensitive to PEDV CHFJFQ, we investigated its infectivity in mice, aiming to evaluate its cross-species transmission capability. Remarkably, our findings show that PEDV CHFJFQ is able to infect mice, targeting primarily the spleen, lungs, and small intestine. Additionally, H&E staining revealed that PEDV CHFJFQ infection led to thickening of the alveolar walls and atrophy of the small intestinal villi in mice. Although PEDV CHFJFQ was found to be enriched in the lungs of mice, it did not cause symptoms in mice, unlike a previous study where neonatal mice infected with PEDV-MK or PEDV-MK-P10 exhibited shivering, growth retardation, and death [61]. These results indicate a limited capacity for cross-species transmission under the tested experimental conditions. However, natural transmission dynamics may differ, and ongoing monitoring is crucial to assess any potential for adaptation and spread to novel hosts. Interestingly, although PEDV CHFJFQ did not infect the liver, it caused liver damage in piglets through an unknown mechanism. While it remains unclear whether PEDV CHFJFQ can be transmitted between pigs and mice, the potential for infection in mice should be considered, as it may represent a potential route in the transmission of PEDV. Therefore, we recommend that pig farms enhance their rodent control measures. Additionally, the potential for PEDV transmission to humans should be considered, as our results demonstrated the sensitivity of human cells to PEDV CHFJFQ. It is crucial to develop appropriate management and prevention strategies to mitigate the risk of cross-species transmission of PEDV to humans.

## 5. Conclusions

In summary, we successfully isolated the CHFJFQ strain from the small intestine of diarrheic pigs and investigated its genetic variation and pathogenicity. This study provides new insights into the prevalence, genetic evolution, and pathogenicity of PEDV. The data contribute to a better understanding of the virus’s origin, genetic recombination, infection characteristics, and potential cross-species transmission. Furthermore, these findings will serve as a valuable reference for future control strategies and the rational design of vaccines targeting PEDV strains circulating in China.

## Figures and Tables

**Table 1:** Nucleotide and amino acid similarity between PEDV CHFJFQ and the bat coronavirus PREDICT/GCS-011.

Gene	PEDV CHFJFQ Strain		PREDICT/GCS-011 Strain (Myotis Bat)		Identity	
	Size (nt)	Size (aa)	Size (nt)	Size (aa)	nt%	aa%
5′UTR	292	-	292	-	96.58	-
ORF1a/b	20,321	6773	20,339	6779	92.02	96.55
S	4149	1382	4128	1357	87.40	90.6
ORF3/NS3	276	91	675	224	93.44	92.59
E	231	76	231	76	93.07	93.42
M	681	226	681	226	93.83	92.92
N	1326	441	1335	444	93.25	94.33
NS7	-	-	390	129	-	-
3′UTR	334	-	248	-	99.55	-
Total	27,953	8989	28,320	9213	91.26	95.4

The results were obtained by Nucleotide BLAST and Protein BLAST in NCBI. Abbreviations: aa, amino acid; nt, nucleotide. The symbol ”-” indicates one of the following: (1) the gene sequence does not encode any amino acids; (2) amino acid similarity is not applicable; or (3) the gene is absent.

## Data Availability

All data generated or analyzed during this study are included in this published article and its Supplementary Information files. The PEDV CHFJFQ genome sequence was deposited in National Center for Biotechnology Information (NCBI), accession number OP688373.

## Supplementary Materials

The following supporting information can be downloaded at https://www.mdpi.com/article/10.3390/v17030401/s1, Figure S1: RT-PCR detection of PEDV and TGEV genomes in intestinal tissues from piglets with diarrhea.; Figure S2: Amino acid deletions, insertions, and substitutions in the CHFJFQ strain compared to the CV777 strain; Figure S3: PEDV CHFJFQ replicated in Vero cells independently of trypsin treatment; Figure S4: The uncropped Western blot image shows the blotting of PEDV-N and GAPDH in Figure 3a; Figure S5: The proliferation of PEDV CHFJFQ in cells was assessed using RT-qPCR and immunofluorescence; Figure S6: Binding and infection of PEDV CHFJFQ in different cells; Figure S7: The uncropped Western blot images in Figure 4; Figure S8: The pathological changes in piglets induced by PEDV CHFJFQ were observed through hematoxylin and eosin (H&E) staining; Table S1: Oligonucleotide primers used for amplification of the complete genome of PEDV CHFJFQ by RT-PCR.; Table S2: The Oligonucleotide primers for RT-qPCR.

## Abbreviations

The following abbreviations are used in this manuscript:

PEDV	Porcine epidemic diarrhea virus
SADS	Porcine acute diarrhea syndrome coronavirus
TGEV	Transmissible gastroenteritis virus
PRRSV	Porcine reproductive and respiratory syndrome virus
PRV	Pseudorabies virus
PCV	Porcine circovirus
DMEM	Dulbecco’s Modified Eagle’s Medium
HEK	Human embryonic kidney
FBS	Fetal bovine serum
PBS	Phosphate-buffered saline
IgG	Immunoglobulin G
hpi	Hour post-infection
dpi	Day post-infection
PCR	Polymerase chain reaction
qPCR	Quantitative polymerase chain reaction
RT-qPCR	Reverse transcription–quantitative polymerase chain reaction
TCID50	50% tissue culture infectious dose
F	Forward
R	Reverse
