# Supplementary material for: The Infectivity and Pathogenicity Characteristics of a Recombinant Porcine Epidemic Diarrhea Virus, CHFJFQ
Source: Viruses. 2025 Mar 12;17(3):401. doi: 10.3390/v17030401 (PMC11945473; doi:10.3390/v17030401)
Supplement: Supplementary file 1 [file viruses-17-00401-s001.zip › viruses-3488364-supplementary.pdf]

Supplementary Figure S1.

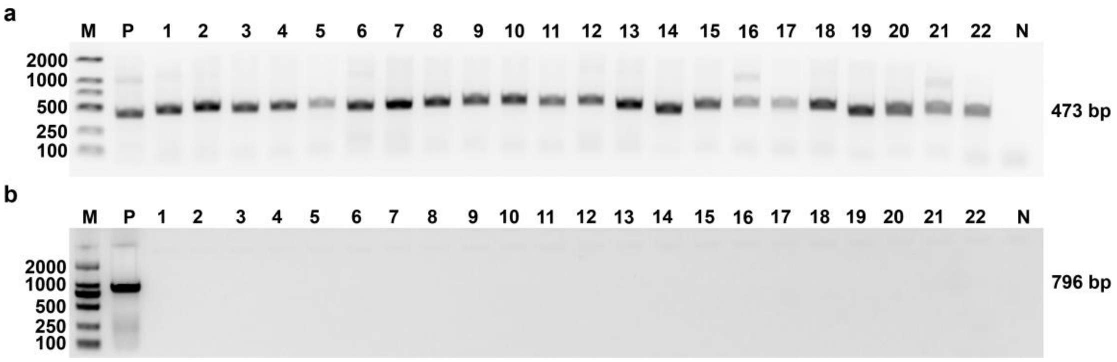

Supplementary Figure S1. RT-PCR detection of PEDV and TGEV genomes in intestinal tissues from piglets with diarrhea. (**a-b**) The PEDV (a) or TGEV (b) S gene was amplified from intestinal tissue cDNA of diarrheal piglets by RT-PCR, and amplification products were visualized by agarose gel electrophoresis. Agarose gels were prepared at 1.5% (w/v) for Supplementary Figure S1a and 1.0% (w/v) for Supplementary Figure S1b. Electrophoresis was performed at 180 V for 12 minutes. The RT-PCR product sizes were 473 bp for the PEDV S gene and 796 bp for the TGEV S gene. The samples were numbered 1-22, with each number representing a unique sample. M indicates the DNA fragment size marker. P and N indicate the positive and negative controls, respectively.

Supplementary Figure S2.

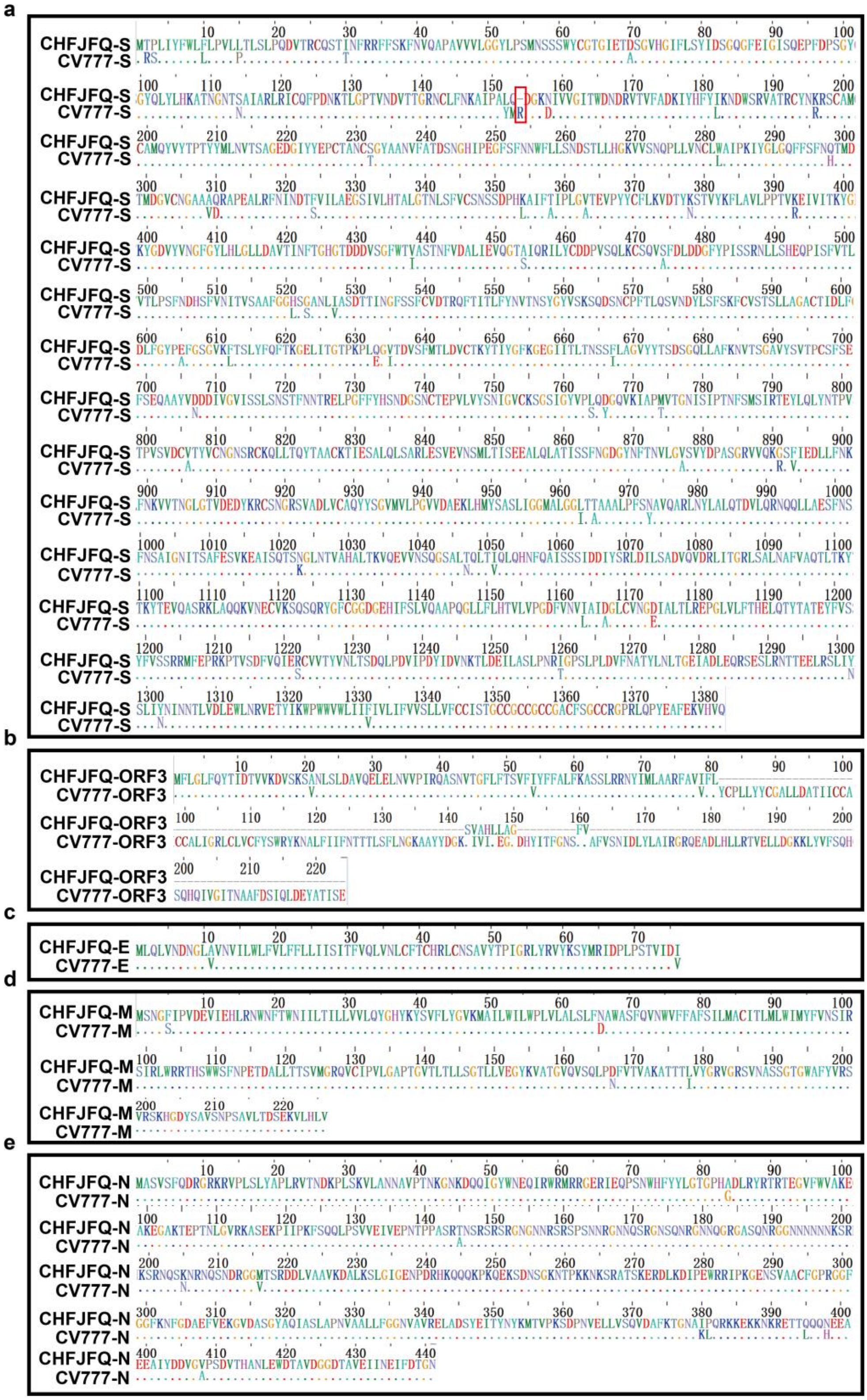

Supplementary Figure S2. Amino acid deletions, insertions, and substitutions in the CHFJFQ strain compared to the CV777 strain. (**a**) The S protein of CHFJFQ exhibited 56 amino acid substitutions and 1 amino acid deletion. (**b**) In the ORF3 protein, CHFJFQ showed 10 amino acid insertions, 3 substitutions, and 143 amino acid deletions. (**c**) The E protein in CHFJFQ displayed 2 amino acid substitutions. (**d**) The

M protein of CHFJFQ had 4 amino acid changes. (**e**) The N protein underwent 9 amino acid changes in CHFJFQ. Sequence alignment was performed using BioEdit software (V.7.0.9.0), employing ClustalW Multiple Alignment (Full Multiple Alignment), with a bootstrap NJ tree set to 1000 bootstraps.

Supplementary Figure S3.

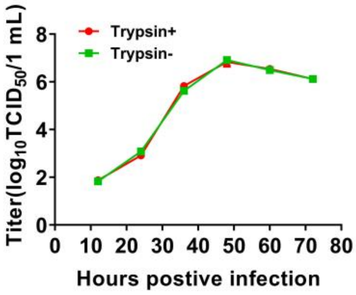

Supplementary Figure S3. PEDV CHFJFQ replicated in Vero cells independently of trypsin treatment. At 48 hours post-infection (hpi), the virus titer in Vero cells reached approximately 1.3×10^7 TCID50/mL. Vero cells (5×10^5 cells per well) were co-cultured with PEDV CHFJFQ (1×10^3 TCID50) in 6-well plates ($n = 3$). Cell culture supernatants were harvested at 12, 24, 36, and 48 hpi, and the virus titer was determined using the Spearman-Kärber method. In the trypsin treatment group, the trypsin concentration was 10 μg/mL.

Supplementary Figure S4.

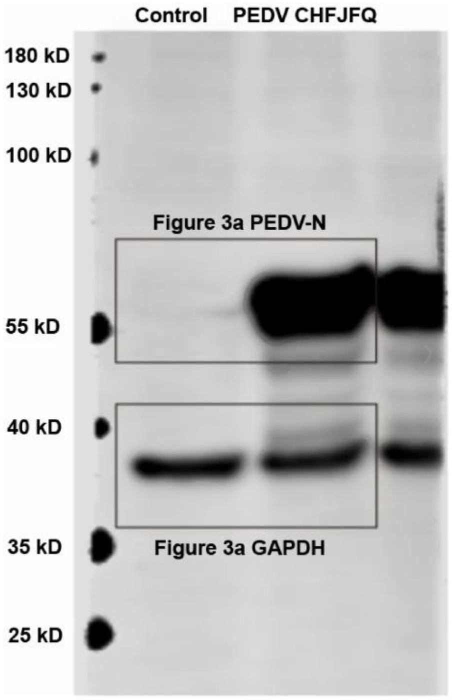

Supplementary Figure S4. The uncropped Western blot image shows the blotting of PEDV-N and GAPDH in Figure 3a.

Supplementary Figure S5.

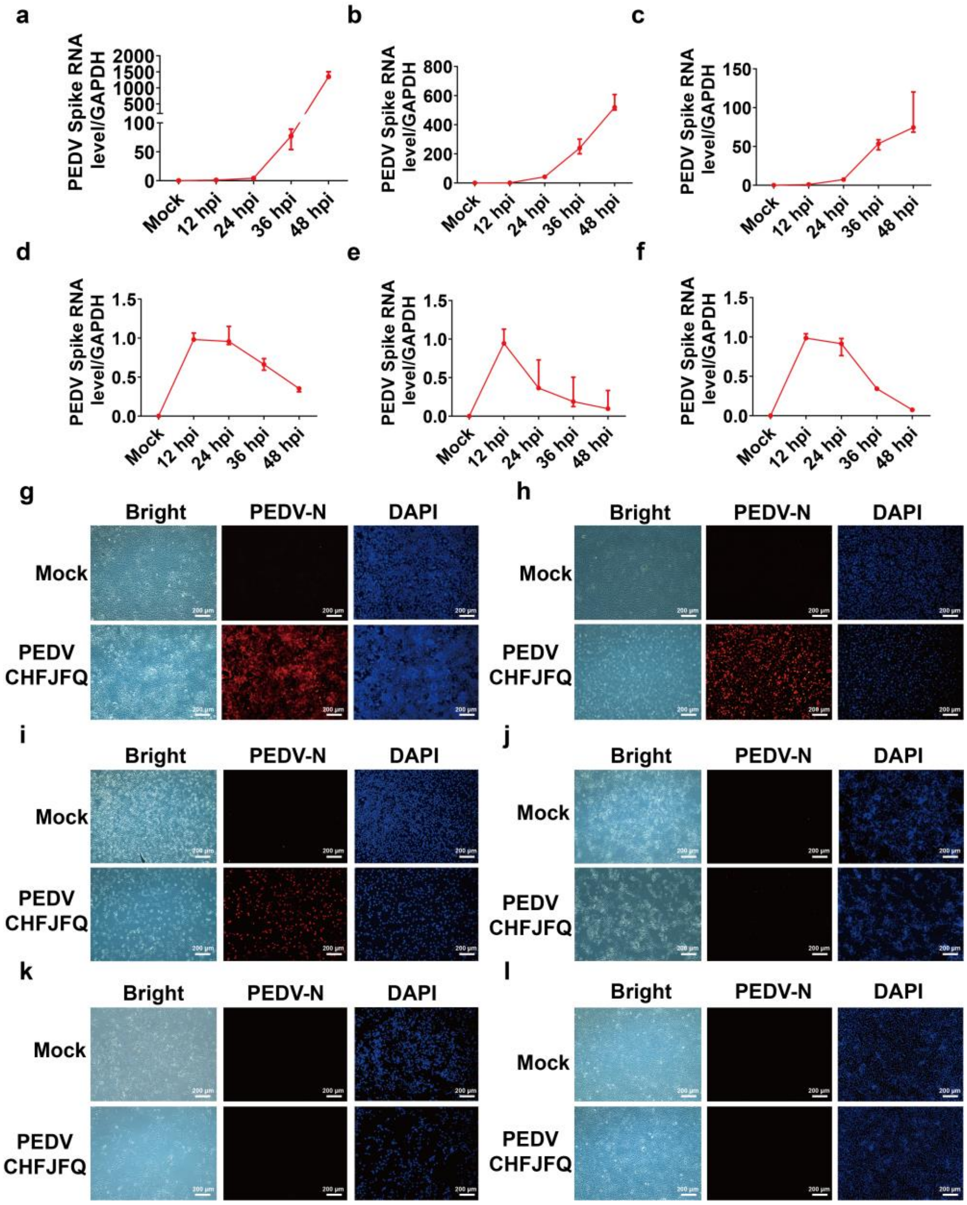

Supplementary Figure S5. The proliferation of PEDV CHFJFQ in cells was assessed using RT-qPCR and immunofluorescence. (**a-f**) The relative levels of the PEDV S gene in 293A, Vero, L929, IPEC-J2, ST, and PK15 cells at different time points post-infection were measured by RT-qPCR (n = 3). Six cell lines (5×10^5 cells per well) were co-cultured with PEDV CHFJFQ (MOI = 0.1) in 6-well plates. Cells were harvested at 12, 24, 36, and 48 hours post-infection (hpi), and total RNA was extracted for RT-qPCR analysis. (**g-l**) The expression of PEDV N protein in 293A, Vero, L929, IPEC-J2, ST, and PK15 cells at 48 hpi was detected by immunofluorescence.

Supplementary Figure S6.

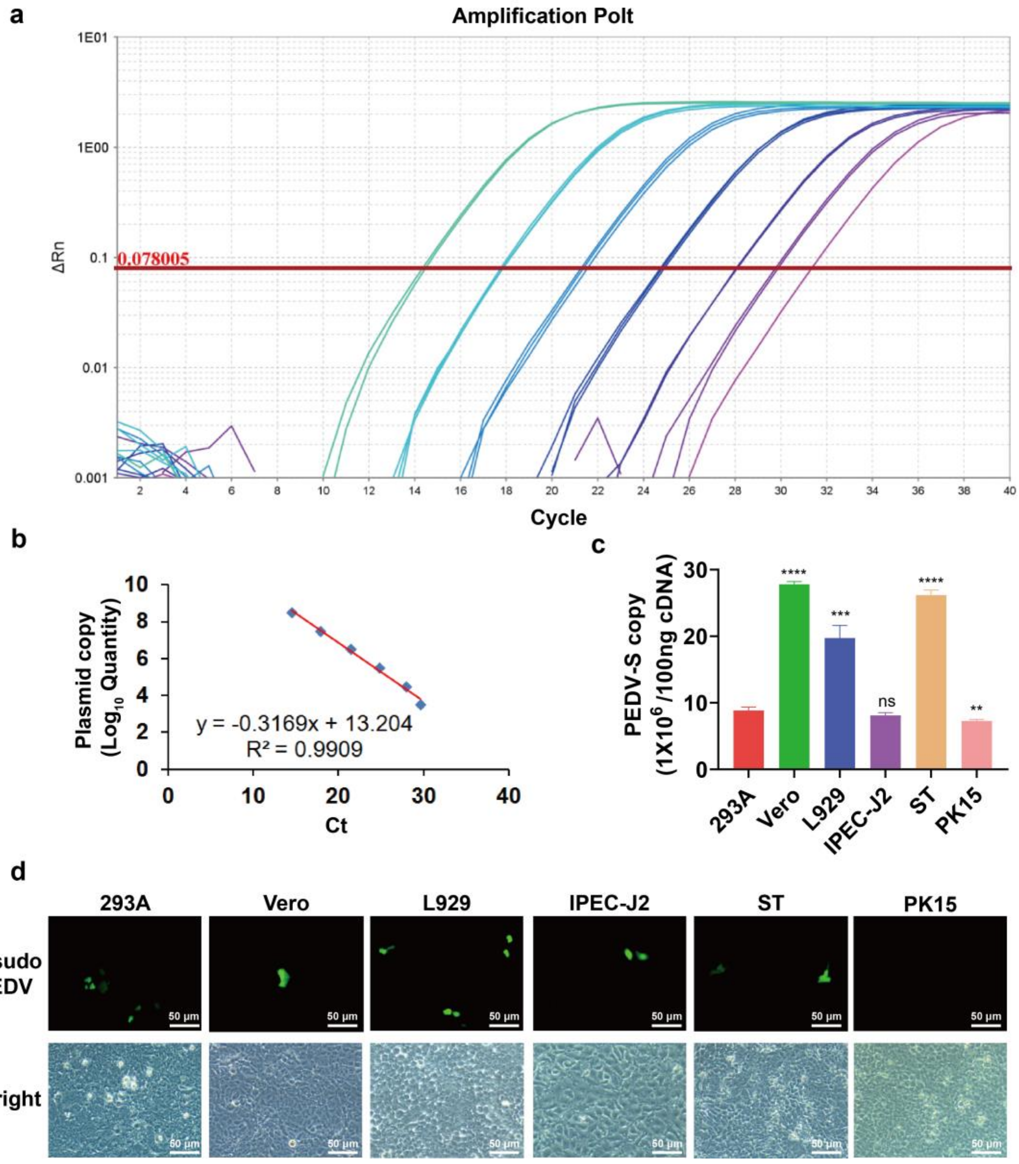

Supplementary Figure S6. Binding and infection of PEDV CHFJFQ in different cells. (**a**) Amplification curves of the plasmid (p19T-S) containing a fragment of the PEDV S gene. (**b**) The standard curve was plotted with plasmid copy (Log10 Quantity) on the ordinate and Ct value on the abscissa. The equation used to calculate the cell surface PEDV CHFJFQ copy is $\log_{10}$ Quantity = −0.3169x + 13.204, where x represents the Ct value. (**c**) The ability of PEDV CHFJFQ strain to bind to cells was determined by the content of S gene nucleic acid on the cell surface (n = 3). (**d**) PEDV CHFJFQ pseudovirus could infect 293A, Vero, L929, IPEC-J2, and ST cells, but not PK15 cells, as indicated by the expression of EGFP. Unpaired t-tests (GraphPad Prism 5.0, GraphPad Software, San Diego, CA, USA) were used to test differences between groups. Data are presented as means ± standard error of the mean for each treatment. **$P < 0.01$, ***$P < 0.001$ and ****$P < 0.0001$ vs. control group (293A), ns: $P > 0.05$ vs. control group (293A). Sample sizes are indicated in brackets.

Supplementary Figure S7.

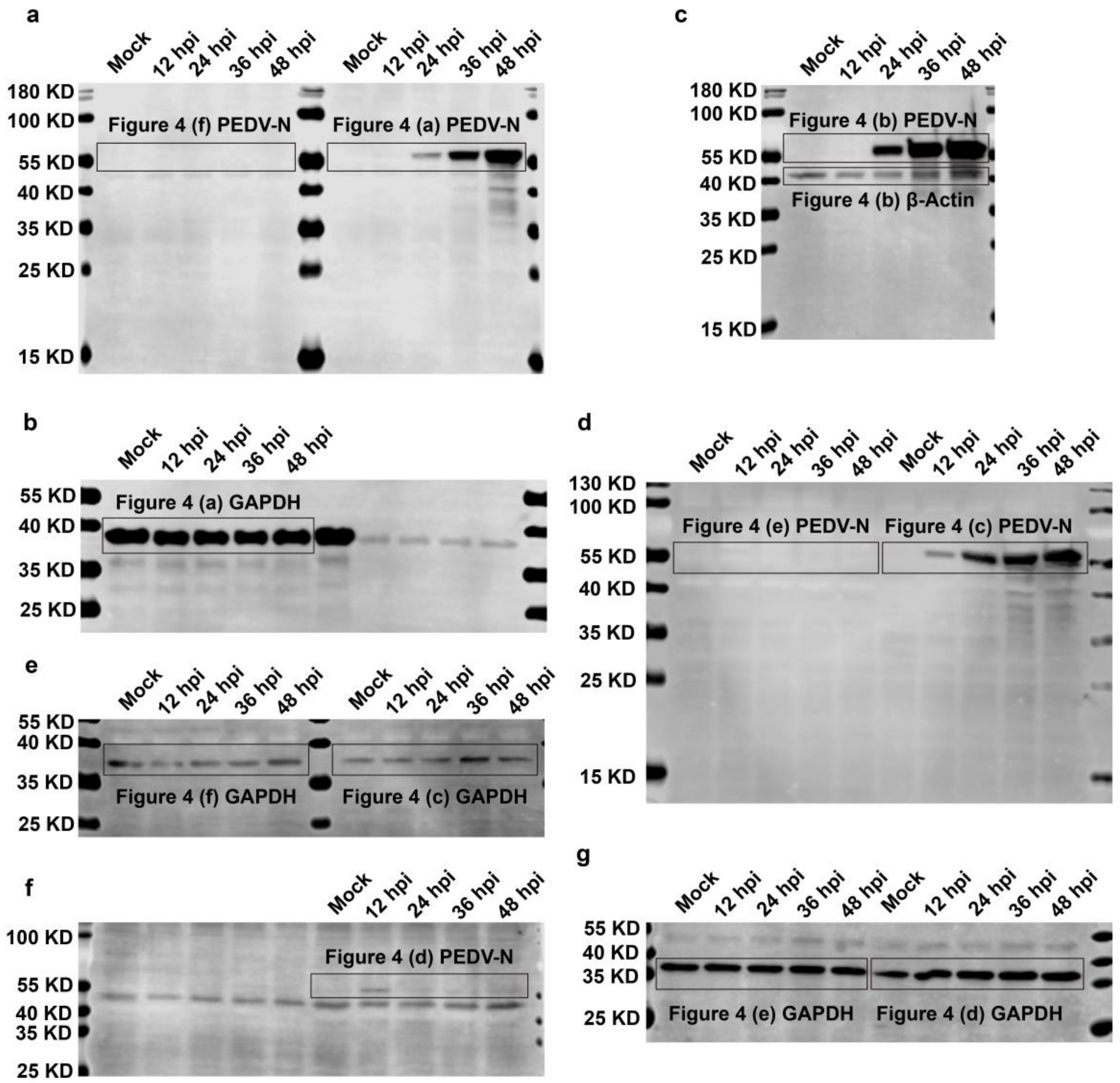

Supplementary Figure S7. The uncropped Western blot images in Figure 4. (**a**) blotting of PEDV-N in Figure 4a and f; (**b**) blotting of GAPDH in Figure 4a; (**c**) blotting of PEDV-N and β-Actin in Figure 4b; (**d**) blotting of PEDV-N in Figure 4c and e; (**e**) blotting of GAPDH in Figure 4c and f; (**f**) blotting of PEDV-N in Figure 4d; (**g**) blotting of GAPDH in Figure 4d and e. These graph represents one of the three repeated experiments.

Supplementary Figure S8.

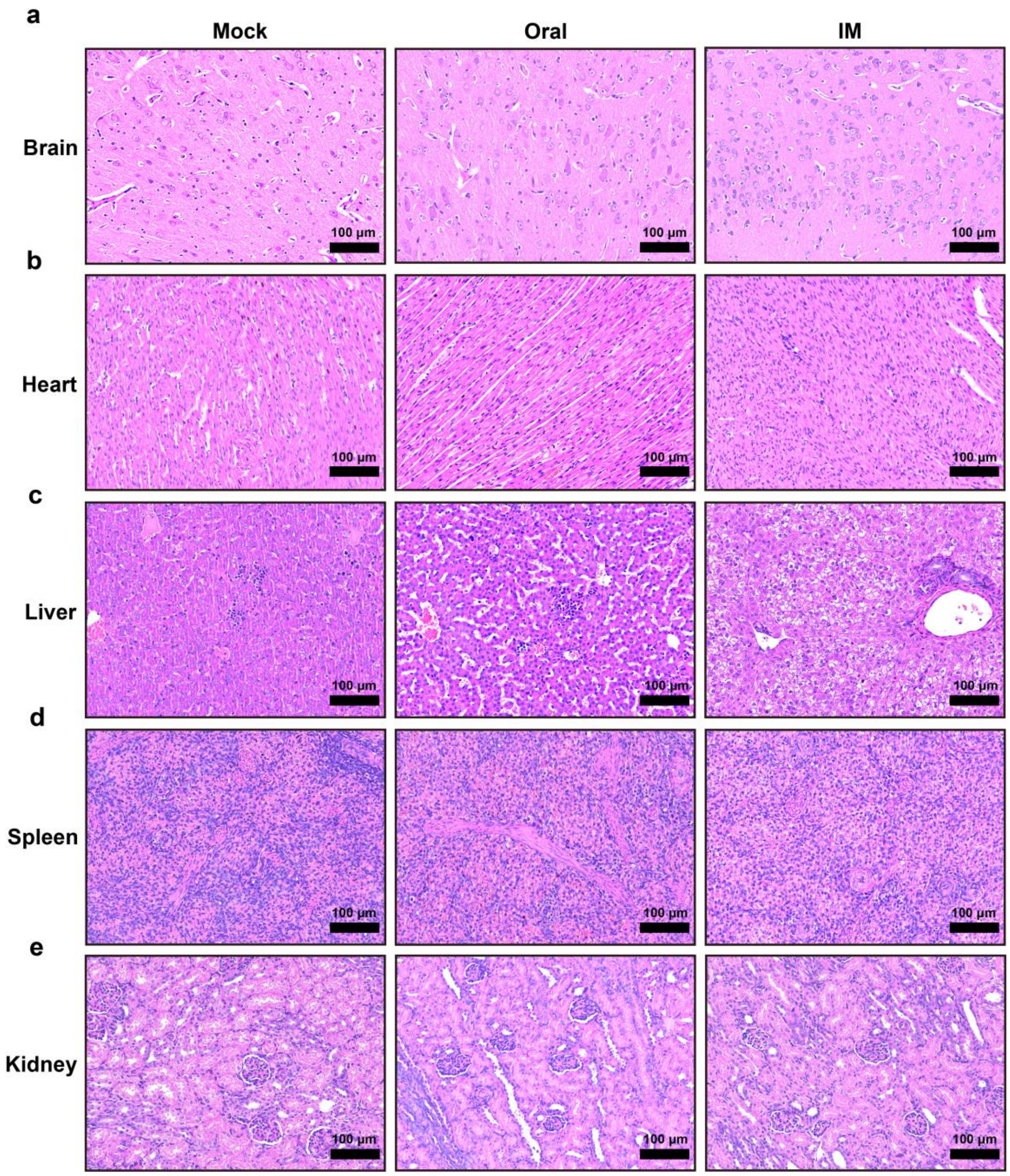

Supplementary Figure S8. The pathological changes in piglets induced by PEDV CHFJFQ were observed through hematoxylin and eosin (H&E) staining. H&E staining was employed to examine the pathological alterations in the brain (**a**), heart (**b**), liver (**c**), spleen (**d**), and kidney (**e**) of piglets following PEDV CHFJFQ infection. These assessments were conducted at 60 hpi.

Supplementary table S1. Oligonucleotide primers used for amplification of the complete genome of PEDV CHFJFQ by RT-PCR.

Primer No.	Primer sequence 5'-3'	Location	Product size (bp)
1-F	ACTTAAAGAGATTTTCTATCTATGGAT	1-27	1150
1-R	CACAACAGAACCAGGCGTA	1132-1150	
2-F	GTTGTCTTGTCTGAGCCACTT	908-928	1307
2-R	TCACCTCTCACCACGCAG	2197-2214	
3-F	GACCCAATACCTGATTTTCCTA	2090-2111	1303
3-R	CCTCAGTTTCAGAGTTAGCCA	3372-3392	
4-F	AATAAGGCTGTTGGTAATCGTA	3083-3104	1551
4-R	AGCATCTACCAAGCCATCC	4615-4633	
5-F	TGTTGCTCTTACACCTTTGATTAG	4468-4491	1063
5-R	CCACATAGCCTGTAAACCTGC	5509-5530	
6-F	TAGACCATACTGGGTTTGCC	5379-5398	1374
6-R	CAAAGACATCAAAAGGCACA	6733-6752	
7-F	TACCAGGAACTCTCGGACTTC	6518-6538	1375
7-R	CAAAGAAAACAGCAACTATGAAC	7870-7892	
8-F	ATTTCATTGCCCTTTCGG	7677-7694	1609
8-R	GGTTGTGCCATCTTACGC	9268-9285	
9-F	GGCGACAAGTTCGTAGGCT	8981-8999	1083
9-R	CAGGGACTGGATTGAGGC	10046-10063	
10-F	TGTTGGTAGCGACTTAGATGGT	9771-9793	1681
10-R	GCCTGTTCCGCCATTCTA	11434-11451	
11-F	AACTTGTGTAATGACCCAGAAA	11123-11144	1278
11-R	GCTCTACAATATAGACACACGGA	12377-12399	
12-F	GGTAAACAAACAGAACAGGCTAT	12167-12189	1391
12-R	AATCCAGCACTTGCGACAC	13539-13557	
13-F	TTACTTGTAGTATCAAGGGAATGG	13214-13237	1378
13-R	GTACCACCTGGCTTCAAATAA	14571-14591	
14-F	AATCCGTGTCTTATGGGTTG	14383-14402	1486
14-R	GTTTGGGTCTGCCGACTT	15851-15868	
15-F	CGATTGGACTGATGTTTCTGA	15684-15704	1641
15-R	AAACGGAAACCCATAAACG	17306-17324	
16-F	CAGACAGTTGATTCATCCCAG	16942-16962	2025
16-R	CGTTCGGCTTCATAGTCCC	18947-18965	
17-F	GGTGCCCACTTCGTTTGAC	18633-18651	1326
17-R	AAGACAGCCGTGCCAGG	19942-19958	
18-F	CAAGCCAGTGAATGGAAGTG	19705-19724	1621
18-R	GCAAATACATTGGCAGCGT	21307-21325	
19-F	AACATGGGATAATGATCGTGTC	21095-21116	1232
19-R	GCAAGGTGAAAGGGCAAT	22309-22326	
20-F	GGAACTGCCATTCAGCGTAT	21960-21979	1477
20-R	CGTCAACAACGCCAGGTAG	23418-23436	
21-F	ATGTGCTGGGTGTTTCCG	23224-23241	1812

21-R	GACTAAACAAAGCCTGCCAAT	25015-25035	
22-F	CTTGGACTTTTTCAATACACGA	24764-24785	1794
22-R	GGCGTGAGGTCCTGTTCC	26528-26545	
23-F	GTTGGGCTTTCTATGTCCG	26182-26200	1772
23-R	GTGTATCCATATCAACACCGTCA	27931-27953	

Abbreviations: F: Forward primer, R: Reverse primer.

Supplementary table S2. The Oligonucleotide primers for RT-qPCR.

**Primer name**	**Sequence (5'-3')**
Pig-GAPDH-F	GGTCGGAGTGAACGGATTT
Pig-GAPDH-R	ATTTGATGTTGGCGGGAT
Mouse-GAPDH-F	AGAGTGTTTCCTCGTCCCG
Mouse-GAPDH-R	GATGGCAACAATCTCCACTTT
Human-GAPDH-F	GGAGCGAGATCCCTCCAAAAT
Human-GAPDH-R	GGCTGTTGTCATACTTCTCATGG
Monkey-GAPDH-F	CAGCCTCAAGATCGTCAGCA
Monkey-GAPDH-R	CGTGGACTGTGGTCATGAGT
PEDV-S-F	CATTTCGCAAGAGCCGTTT
PEDV-S-R	TTGTTGAATAGGCAGTTACGACC
PEDV-N-F	TGAGGGTGTTTTCTGGGTTG
PEDV-N-R	TTGCCATTGCCACGACTC

Abbreviations: F: Forward primer, R: Reverse primer.
